# Impact of sequencing read quality on whole-genome sequencing outcomes for foodborne pathogens using a BacWORK standardized analysis workflow

**DOI:** 10.1093/bioadv/vbag130

**Published:** 2026-05-07

**Authors:** Arnaud Felten, Deborah Merda, Meryl Vila Nova, Fabrice Touzain, Virginie Chesnais

**Affiliations:** ANSES, Ploufragan-Plouzané-Niort Laboratory, VIRPIG Unit, Ploufragan F-22440, France; University Paris Est, ANSES, Laboratory for Food Safety, SPAAD Unit, Maisons-Alfort F-94701, France; University Paris Est, ANSES, Laboratory for Food Safety, SPAAD Unit, Maisons-Alfort F-94701, France; ANSES, Ploufragan-Plouzané-Niort Laboratory, VIRPIG Unit, Ploufragan F-22440, France; University Paris Est, ANSES, Laboratory for Food Safety, SPAAD Unit, Maisons-Alfort F-94701, France

## Abstract

**Motivation:**

Whole-genome sequencing (WGS) is now routinely used for surveillance and outbreak investigations of foodborne pathogens. However, the reliability of downstream analyses strongly depends on sequencing read quality, including depth of coverage and the presence of contamination. Systematic evaluation of how these factors affect WGS outputs remains essential to ensure robust, reproducible, and interpretable results.

**Results:**

We evaluated the impact of sequencing depth and read quality on WGS-derived analyses of foodborne pathogens using an automated, reproducible workflow. Artificial Illumina paired-end datasets were generated for 11 bacterial pathogens with varying depths of coverage (25×–500×) and controlled quality alterations, including intra- and inter-species contamination. Low sequencing depth resulted in fragmented and incomplete genome assemblies, leading to reduced performance in downstream typing analyses. While contamination impacted assembly fragmentation, it did not compromise completeness. However, it caused significant errors in both serotyping and cgMLST analyses. Application to real outbreak datasets showed that the introduction of as little as 5%–10% contamination altered a *Salmonella enterica* strain clustering by increasing cgMLST allelic distances with epidemiologically related isolates. These findings highlight that even low-level contamination can significantly compromise bacterial typing and outbreak interpretation. The BacWORK workflow was used to support this evaluation and to demonstrate compliance with NF EN ISO 23418 for quality-aware WGS analysis.

**Availability and implementation:**

The BacWORK workflow is available at https://github.com/FBi-ANSES/BacWORK.

## 1 Introduction

Whole-genome sequencing (WGS) is now the gold standard for the analysis of bacterial isolates, and has been extensively employed in both fundamental research and public health for the surveillance and monitoring of pathogens. Using the state-of-the-art WGS technique, bacterial draft genomes can be obtained within a few hours or days, and data analysis has become a routine task for most laboratories. Over the last few years, it has been commonly admitted that single nucleotide polymorphism (SNP) or core-genome multi-sequence locus sequence typing (cgMLST) enables an accurate resolution for comparing and characterizing microbial pathogens (Moura *et al.* 2016, Gerner-Smidt *et al.* 2019, Timme *et al.* 2020). Indeed, high-resolution WGS is used for outbreak investigation and monitoring, providing valuable subtyping information such as serotype, and virulence or resistance gene profiles. Several initiatives have proven the success of using WGS in national laboratory surveillance programs, including the GenomeTrackr database (Timme *et al.* 2021) or the project proposed by Jackson *et al.* (2016) that aimed to make available every *Listeria monocytogenes* genome obtained from US food supplies.

Many workflows have been developed by the scientific community to extract information and gather data from WGS. These include algorithms for raw read quality control (Bolger *et al.* 2014, Chen *et al.* 2018), bacterial genome assembly (Wick *et al.* 2017, Seemann 2017, Prjibelski *et al.* 2020), and strain typing using MLST based on either seven housekeeping genes or core-genome approaches (Clausen *et al.* 2018, Silva *et al.* 2018). Standard downstream analysis tools are also available for tasks such as serotyping, antimicrobial resistance (AMR) gene detection, and de novo genome annotation (Bortolaia *et al.* 2020, Schwengers *et al.* 2021, Liu *et al.* 2022). The production of increasingly large amounts of data through WGS, coupled with the progress of sequencing technology, has led to an ever-growing library of bioinformatics tools dedicated to sequencing analysis. Nonetheless, the field is still lacking standardized, reproducible workflows that allow comparison over time and between datasets. Indeed, only a handful of functional workflows have taken consistency between analyses into consideration, including Nullarbor (Seemann 2015), Bactopia (Petit and Read 2020), AQUAMIS (Deneke *et al.* 2021), TORMES (Quijada *et al.* 2019), ASA3P (Schwengers *et al.* 2020), rMAP (Sserwadda and Mboowa 2021), and CABGen (Duré *et al.* 2022). These were all developed around package managers [Mamba (Quant Stack and Contributors 2025), Anaconda (Grüning *et al.* 2018), Brew (yugabyte 2018), software containers [Docker (Docker Documentation) or Singularity (Singularity container Documentation)], and workflow management tools [Snakemake (Mölder *et al.* 2021) or Nextflow (Di Tommaso *et al.* 2017)]. However, most of the workflows currently implemented were designed to be generic, and are generally restricted to assembling reads after quality filtering, without taking into account species-specific analyses such as serotyping or other dedicated typing schemes. Most of them do not include data gathering steps to retrieve key information from the sequencing data such as virulence genes, mobilome characterization, or phage detection. Moreover, they lack steps needed to characterize and type a number of specific foodborne pathogens of importance monitored by public health agencies.

The reproducibility of bioinformatics analyses is improved by the development of automated pipelines that process all the analysis steps, from raw data to annotated genomes. However, deterministic variations in algorithms can skew analysis results, as can fluctuating quality thresholds (Baykal *et al.* 2024). For example, it has been shown that replications of bioinformatics analysis on the same sequencing results can lead to different cgMLST profiles (Merda *et al.* 2024). Moreover, both poor read quality and sequencing depth impact cgMLST results, leading to variations in observed allelic distance between strains (Merda *et al.* 2024). Additionally, intra-species and inter-species contamination at high levels has been shown to modify SNP clustering among strains (Pightling *et al.* 2019). Consequently, the quality of data and results must be fully under control to ensure validity of downstream analysis and enable data exchange between public health agencies. While many new tools have been developed in recent years, there are too few studies that evaluate these tools on compromised WGS data, particularly during some of the analysis steps, such as annotation or typing.

To systematically assess how sequencing quality constraints influence whole-genome sequencing (WGS) analyses of foodborne pathogens, we conducted a comprehensive evaluation using BacWORK, an integrated bioinformatics workflow designed for flexible analysis of sequencing data on foodborne bacterial pathogens. Implemented with a combination of Snakemake and Mamba, BacWORK is a scalable workflow that can manage the analysis of any number from a couple to a few thousand isolates simultaneously with limited configuration needs and a simple single command line trigger. This workflow offers many different outputs that are easily compiled for a global quality report but that can also be used for additional downstream analyses (pan-genomic, phylogenies, etc.). For versatility, we integrated as many species-specific typing approaches as possible to include all the pathogens involved in foodborne outbreaks. To meet the needed quality threshold for WGS interpretation, BacWORK was validated on various datasets, including both high- and low-quality data on 11 different species or subspecies (*Bacillus cereus*, *Bacillus thuringiensis*, *Campylobacter jejuni*, *Clostridioides difficile*, *Clostridium perfringens*, *Escherichia coli*, *L. monocytogenes*, *Salmonella enterica*, *Staphylococcus aureus*, *Vibrio cholera*, and *Vibrio parahaemolyticus*). We demonstrates how sequencing depth and contamination affect WGS interpretation and shows that a standardized, quality-aware workflow can provide robust and reproducible results for outbreak investigations.

## 2 Methods

### 2.1 Implementation of BacWORK

**Figure 1:**
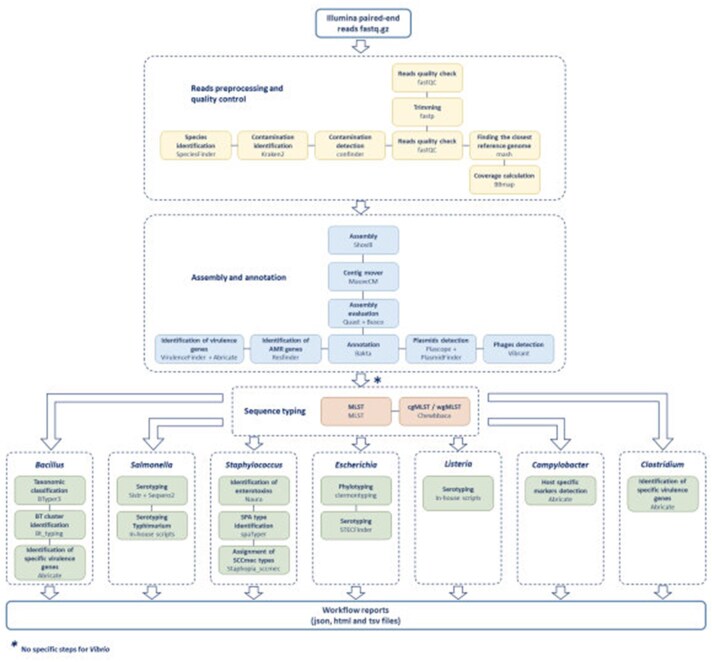
Overview of BacWORK. Preprocessing of raw reads to assess quality and find the closest available reference ("Reads preprocessing and quality control" block). Based on the trimmed reads, contigs are assembled and annotated with several tools ("Assembly and annotation" block). Sequence typing is performed on seven genes and the core genome ("Sequence typing" block). Additional typing tools, specific to species, are also available to better characterize bacterial strains (species blocks). The results are presented in structured TAB and JSON files, and reports are displayed in HTML format.

BacWORK is an automated Snakemake-based workflow designed for the primary and secondary analysis of bacterial paired-end Illumina sequencing data that inherently documents workflow steps, inputs, and outputs. To ensure reproducibility, facilitate deployment and prevent issues related to missing or mismatched dependencies, it uses Mamba environments for each analysis step. The workflow is structured into modules that are either common or pathogen-specific. Modules, tools, and databases are provided in Table 4, available as supplementary data at *Bioinformatics Advances* online and depicted in Fig. 1. The versions listed in the tables, available as supplementary data at *Bioinformatics Advances* online, and manuscript correspond to those used in our study for workflow evaluation and validation.

The first module is dedicated to the preprocessing of reads: Fastp v0.23.4 (Chen *et al.* 2018) is used for trimming and filtering raw reads and fastQC v0.12.1 (Leggett *et al.* 2013) is used to check read quality before and after filtering. The critical step of species identification is performed with SpeciesFinder v1.5.6 (Larsen *et al.* 2014), and results are used at different levels throughout the analysis. Contamination is evaluated with ConFindr v0.8.1 (Low *et al.* 2019) using the rMLST schema for intra-species contamination. Kraken2 v2.1.3 (Wood *et al.* 2019) is used to check inter-species contamination by comparing any identified genus and species that differ from species identified by SpeciesFinder. Finally, the closest reference genome is found using Mash v2.3 (Ondov *et al.* 2019) against in-house databases constructed using all available complete genomes of the studied species downloaded from the NCBI using ncbi-genome-download v0.2.8 (Blin 2023), and sequencing coverage and depth is evaluated with BBmap v39.06 (Bushnell 2014).

The second module is dedicated to assembling and annotating the bacterial genome. Shovill v1.1.0 (Seemann 2017) is used to assemble genomes and the resulting contigs are reordered using MauveCM v2.4.0 (Rissman *et al.* 2009). Small contigs are filtered out, and assembly quality is assessed both by mapping reads back to the contigs and by comparing the assembly against the closest reference genome previously identified, using QUAST v5.2.0 (Gurevich *et al.* 2013). Completeness of assembly is checked using BUSCOs v5.7.1 (Simão *et al.* 2015). The resulting assembly is annotated by Bakta v1.9.3 (Schwengers *et al.* 2021). Virulence is annotated using ABRicate v1.0.1 (Seemann 2020) and the VFDB database (Liu *et al.* 2022), while resistance is annotated using ResFinder v4.5.0 (Bortolaia *et al.* 2020) directly on trimmed and filtered reads. Plascope v1.3.1 (Royer *et al.* 2018) and PlasmidFinder v2.1.6 (Carattoli *et al.* 2014) are used to detect plasmids, and Vibrant v1.2.1 (Kieft *et al.* 2020) is used to search for phages in assemblies.

The last shared module is used to type bacteria: MLST is determined using MLST v2.23.0 (Seemann 2010) and cgMLST is computed with chewBBACA v3.1.2 (Silva *et al.* 2018) by looking for core-gene alleles in the assembly. To ensure interoperability between laboratories and remove dependence on a single centralized database, allele sequences are hashed using an in-house script in accordance with EFSA recommendations. The list of cgMLST schemas used in this study is provided in Table 5, available as supplementary data at *Bioinformatics Advances* online, and chewBBACA schemas were prepared with default parameters. Spanning tree was constructed from the cgMLST allele matrix using GrapeTree (Zhou *et al.* 2018).

Finally, BacWORK enables additional species-specific genome annotation. For *Bacillus* genera, taxonomic classification, Bt cluster identification and the search for specific virulence genes are performed, respectively, using BTyper3 v3.4.0 (Carroll *et al.* 2020), the Bt_typing in-house script (Fichant *et al.* 2022), and ABRicate v1.0.1 using an in-house database. *Salmonella* is serotyped with Sistr v1.1.2 (Yoshida *et al.* 2016) and SeqSero2 v1.3.1 (Zhang *et al.* 2015), while the serotyping of specific Typhimurium variants is assessed using isPCR v33 and in-house scripts to detect mdh, fliC, fljB, and fliA genes. This step was validated using sequencing results obtained from 15 strains characterized through classical serotyping approaches. *Staphylococcus* enterotoxins are identified using Naura v0.5 (Merda *et al.* 2020), spa type is identified with spaTyper v0.3.3 (Bartels *et al.* 2014) and SCCmec types are assigned with Staphopia SCCmec v1.0.0 (Petit and Read 2020). *Escherichia* phylotyping and serotyping are performed with ClermonTyping v1.0.0 (Beghain *et al.* 2018) and STECfinder v1.1.0 (Zhang *et al.* 2022), respectively. For *Listeria*, the serotype is determined using isPCR v33 and an in-house script to detect prfa, lmo1118, lmo0737, ORF2819, ORF2110, and prs genes. This step was validated using 10 public genomes with various reported serotypes (IIa: SRS26168545, SRS26168560, ERS26933675; IIb: SRS26168540, SRS26168535, ERS26933657; IIc: SRS26168534, SRS26168495; IVb: ERS26933753, ERS26933755). *Campylobacter*- and *Clostridium-specific* biomarkers are predicted using ABRicate and in-house databases. The versions of the databases used in this study are detailed in Tables 4 and 5, available as supplementary data at *Bioinformatics Advances* online. As differences in database versions can influence results, users should take this into account when interpreting or comparing analyses.

### 2.2 Versioning and quality of workflow

Versioning is a system that tracks and documents changes in code or datasets, enabling transparency, error correction, and the coexistence of stable and customized workflows. It allows bioinformaticians to develop, test, and update workflows without disrupting access to validated versions. Using version control ensures clear documentation and facilitates any rollback of changes, fostering cooperation and innovation. Auditability ensures that workflows record detailed reports of their processes, including software versions, data inputs, and settings. Automatically storing intermediate files in standardized formats (e.g. FASTA, CSV, JSON) aids troubleshooting by identifying discrepancies and their origins. This transparency facilitates reproducibility and error resolution.

The BacWORK workflow was developed in line with FAIR principles (Findable, Accessible, Interoperable, Reusable) through the use of Git, Snakemake, and Mamba. Each tool operates within its own dedicated Mamba environment, where specific versions have been predefined to ensure reproducibility. Tool parameters, database paths, and quality thresholds can be easily adjusted by modifying configuration files in JSON format. Furthermore, to comply with the NF EN ISO 23418 standard on whole-genome sequencing for the typing and genomic characterization of bacteria in the food chain, traceability files are generated for each analyzed sample. These files document the tool versions, their configurations, and the corresponding database versions, ensuring full transparency and reproducibility. The BacWORK workflow is freely available at https://github.com/FBi-ANSES/BacWORK.

### 2.3 Dataset used for validation

To validate the workflow, we simulated two types of sequencing datasets. For five to eight different reference genomes among our selection of 11 pathogenic foodborne bacterial species (*B. cereus*, *B. thuringiensis*, *C. jejuni*, *C. difficile*, *C. perfringens*, *E. coli*, *L. monocytogenes*, *S. enterica*, *S. aureus*, *V. cholerae*, and *V. parahaemolyticus*), we generated sequencing paired-end reads at six depths: 25×, 50×, 75×, 100×, 250×, and 500×. The genome simulation method was that of a previous study (Merda *et al.* 2024), and all the samples are listed in Table 6, available as supplementary data at *Bioinformatics Advances* online. To assess contamination detection and its impact on results, we artificially contaminated the previous 100× simulated dataset with both intra-species and inter-species reads at contamination levels ranging from 1% to 15%. Contaminated datasets were generated randomly, with contamination levels and source species randomly selected. For intra-species contamination, reads were selected from other available genomes of the same species, whereas inter-species contamination included reads from any of the other species’ genomes. This approach enabled us to simulate a total of 1450 paired-end read files with intra-species contamination and 1450 paired read files with inter-species contamination. A full description of contaminated samples is provided in Table 7, available as supplementary data at *Bioinformatics Advances* online, and the distribution of randomly picked ratios of contamination is represented in Fig. 1, available as supplementary data at *Bioinformatics Advances* online. All simulated datasets (i.e. reads generated in silico) are available on demand (>1.5 TB). The scripts used to generate the simulated datasets are publicly available on GitHub (https://github.com/FBi-ANSES/BacWORK_validation), allowing readers to reproduce the simulations or generate subsets of the data as needed.

The workflow was also evaluated on the EURL datasets, available on demand (Inter-EURLs Working Group on NGS 2023), for *C. jejuni*, *E. coli*, *L. monocytogenes*, and *S. enterica*. We were able to analyze nine replicates of one *E. coli* strain, between 7 and 17 replicates of four *C. jejuni* strains, between two and three replicates of four *L. monocytogenes* strains and between 13 and 14 replicates of six *S. enterica* strains.

Outbreak data were obtained from published datasets and downloaded from the Sequence Read Archive (SRA) according to authors’ recommendations. We downloaded 23 paired-end reads from an outbreak involving *L. monocytogenes* (Halbedel *et al.* 2023)and 34 paired-end reads from an outbreak involving *S. enterica* serotype Welikade (Cherchame *et al.* 2022) prior to BacWORK analyses.

## 3 Results

### 3.1 Overview of BacWORK

BacWORK is dedicated to the analysis of paired-end Illumina reads obtained from WGS of bacterial isolates. The workflow is described in Fig. 1. Compared with other available workflows described in Table 1, available as supplementary data at *Bioinformatics Advances* online, BacWORK guarantees, with AQUAMIS, the most robust raw data quality check and quality filtering available by taking into account inter- and intra-species contamination. The resulting filtered high-quality reads are assembled using a default tool [i.e. Shovill (Seemann 2017)] proven to be efficient for most foodborne pathogens in a recently published benchmark (Merda *et al.* 2024). To this we added a contig reordering step based on most-related reference genome. Like other workflows, BacWORK is capable of MLST typing, but we also added cgMLST to provide accurate typing tools, as this is currently the most widely used approach for outbreak investigations. Beyond classical de novo annotation of a generated assembly and screening for virulence and resistance genes, we went one step further in annotating genomes by searching for plasmid and prophage sequences. The biggest advantage of BacWORK lies in its modular species-specific typing, which provides state-of-the-art serotyping steps for each pathogen. For example, BacWORK offers specific *S. enterica* typhimurium variant serotyping, thus helping to increase the accuracy of results; *L. monocytogenes* serotyping using in-house scripts; clustering and serotyping of Shiga toxin-producing *E. coli* (STEC) strains, or spa typing and enterotoxin identification for *S. aureus*.

**Table 1:** Impact of low depth on typing approaches.^a^

Species	Typing method	Total samples	Errors in 25×	Errors in 50×
**All**	ST	93	54	2
***Bacillus cereus***	Group	16	1	0
***Staphylococcus aureus***	Spa typing	9	2	0
***Salmonella enterica***	Seqsero2	11	4	0
***Salmonella enterica***	SISTR	11	3	0

a Simulated 25× and 50× depth samples were typed and the results were compared against controls. Errors in sequencing typing results are displayed.

### 3.2 Identifying low-quality sequencing samples using multiple genomic metrics

To evaluate the requested quality threshold for high-accuracy WGS analysis and guarantee the stability of results obtained from these data, we simulated datasets of various qualities. Using five to eight reference genomes for our selected 11 species or subspecies involved in foodborne outbreaks, we simulated sequencing results with a coverage depth of 100× as a sequencing control. To investigate the impact of lower and higher sequencing depths, we also simulated raw read datasets with coverage depths ranging from 25× to 500×. Datasets of 2900 in silico samples with simulation of intra-species and inter-species contamination were also produced by mixing reads from a target reference with reads obtained from the same or another species, and at a different random ratio between 1% and 15% (Fig. 1, available as supplementary data at *Bioinformatics Advances* online).

Samples sequenced at an insufficient depth are easily identified with BacWORK by computing the depth and breadth of coverage through the alignment of reads to the closest matching reference genome. Depth and breadth coverage were used as a quality control feature in BacWORK.

**Figure 2:**
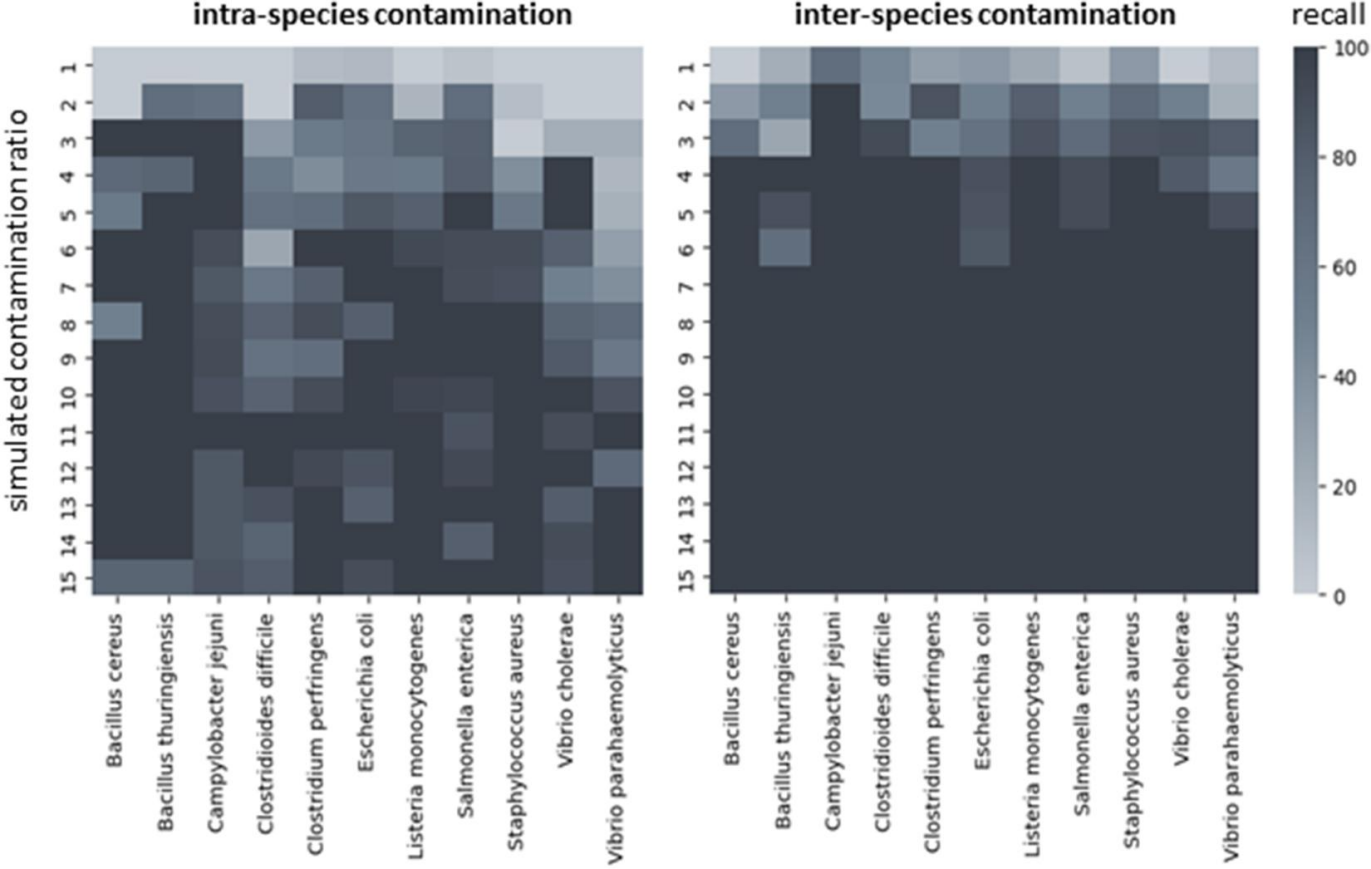
Detection recall of intra- and inter-species contamination by the ConFindr and Kraken2 reference tools. Evaluation of simulated intra- and inter-species contamination by BacWORK through computing recall (i.e. sensitivity) of detection using both ConFindr and Kraken2 tools. Intra-species contamination is on the left and inter-species contamination on the right. The sample detection recall is given for each species and simulated contamination ratio.

As expected, intra-species contaminations were only detected by ConFindr, since Kraken2 was not developed for this purpose. The median number of SNVs detected from contaminated intra-species samples was 12 (range: 0–168 SNVs; mean: 23 SNVs). Even though the number of SNVs differed for each species (with a median of four SNVs for *V. parahaemolyticus* and up to 40 SNVs for *C. jejuni*, *B. cereus*, and *B. thuringiensis*), a correlation was always observed between SNVs and the contamination ratio (Pearson *r*: 0.60–0.80) (Fig. 2, available as supplementary data at *Bioinformatics Advances* online). With a threshold of three SNVs as recommended in the ConFindr documentation, we were able to identify 1095 (76%) of all contaminated samples with a single occurrence of a false positive reported in negative controls for *C. jejuni*. We observed species-specific variations with, for example, high confidence in ConFindr results for both *Bacillus* species, but poor contamination detection in *C. difficile* or *V. parahaemolyticus*. Precision of ConFindr was satisfactory, with very few false positives observed in the uncontaminated controls used in the study (FPR ranging from 12.50 to 0.0, median 0.0). Overall, ConFindr was able to detect most of the intra-species contaminations but not all, even at a high ratio of contamination (Fig. 2).

On the other hand, ConFindr did not perform well in detecting inter-species contamination, identifying only 422 (29%) of contaminated strains. The median number of SNVs detected was one (range: 0–418 SNVs; mean: 27 SNVs). Kraken2 performed significantly better, detecting 1272 (88%) inter-species contaminations with few false positives. When focusing on contamination ratios above 2%, Kraken2 identified 1211 (96%) contaminated samples, with minor differences between species. In particular, for *B. cereus* and *B. thuringiensis*, detection rates were slightly lower, standing at 90% and 96%, respectively. The integration of both ConFindr and Kraken2 further improved contamination detection, identifying 20 additional contaminated samples, including up to eight *Clostridioides difficile* samples across various contamination ratios. This combined approach resulted in a single false positive among 93 negative controls in *C. jejuni*, which still enables a high precision in contamination detection (FPR ranging from 12.50 to 0.0, median 0.0). Implementation of both Kraken2 and ConFindr enables most inter-species contaminations to be detected, especially for contamination ratios over 2% (Fig. 2).

Finally, considering both types of contamination, integrating ConFindr and Kraken2 offers the possibility of identifying a contamination with a recall ranging from 68% for *V. parahaemolyticus* to 88% for *B. thuringiensis*. For contamination ratios greater than 2%, recall is significantly better, ranging from 79% for *V. parahaemolyticus* to 98% for *B. thuringiensis*.

### 3.3 Assembly completeness depends on sequencing depth and the contamination status of sequencing data

**Figure 3:**
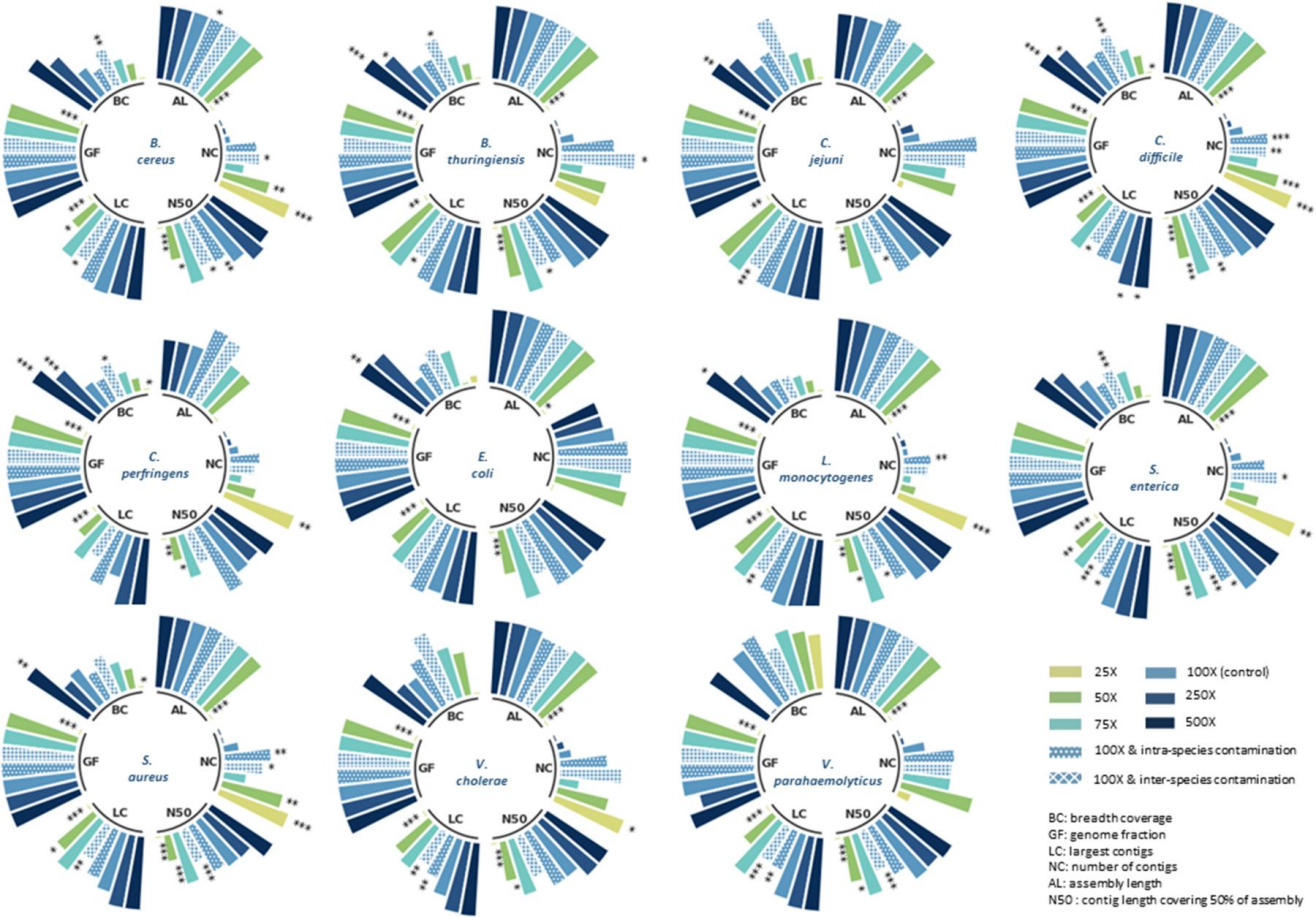
Impact of the coverage depth and contamination of raw reads on assemblies. Simulated reads with various depths and contamination types and levels were assembled with BacWORK. The impact of read quality on assemblies was evaluated using Breadth Coverage (BC), Genome Fraction (GF), Largest Contig Length (LC), N50, Number of Contigs (NC), and Total Length of Assembly (AL) metrics. For the plots, the median for each species was scaled using the min-max scaler method. All conditions were compared against the non-contaminated 100× dataset used as a control using the Student’s *t*-test.

To further characterize the impact of poor-quality samples due to a low depth of coverage and/or contamination, and to define relevant quality thresholds, we investigated their impact on BacWORK results. At low depths, we observed a significant increase in fragmentation and a decrease in assembly length for most of the species studied. Indeed, genome fraction, longest contig length, N50, and total assembly length were significantly different at 25× and 50× when compared with 100× controls for most species (Fig. 3). BUSCO scores were found to be significantly lower when the depth of coverage was low, meaning that shorter and more fragmented assemblies were also less complete (data not shown). On the other hand, higher coverage depths (250× and 500×) slightly improved the quality of assemblies, with a greater breadth of coverage of the closest reference in 3/11 and 7/11 species at 250× and 500×, respectively.

Overall, inter-species contamination was observed to impact genome assemblies, as significant differences were observed in assembly fragmentation, with variations found in breadth coverage, longest contig length, contig number and N50 for 5/11 to 9/11 species (Fig. 3). However, the completeness of assembly was not impacted as no differences were observed for duplicated, fragmented, or missing BUSCO marker genes for 10/11 species. When considering the contamination ratio, we found a moderate correlation between contamination ratio and assembly fragmentation as the number of contigs increases (*R*^2^ = 0.26) and N50 decreases with the simulated contamination ratio (*R*^2^ = −0.37) (Fig. 3, available as supplementary data at *Bioinformatics Advances* online). Additionally, inter-species contamination significantly influenced the percentage of reads mapped back to the assembly when comparing contaminated and uncontaminated samples (*P* value <.01; median: 91% vs. 99%). A negative correlation was observed between the percentage of reads mapped and the contamination ratio for all species (*R*^2^ ranging from −0.79 and −0.91).

Unlike inter-species contamination, intra-species contamination did not greatly affect genome assemblies, as few differences were observed between contaminated and control assemblies in 1/11 to 3/11 species (Fig. 3). Completeness of assembly was not impacted as no differences were observed for duplicated, fragmented, or missing BUSCO marker genes for 9/11 species. Finally, intra-species contamination slightly influenced the percentage of reads mapped back to the assembly when comparing contaminated and uncontaminated samples (*P* value <.01; median: 98% vs. 99%). A negative correlation was observed between the percentage of reads mapped and the contamination ratio for most but not all species (*R*^2^ ranging from −0.23 and −0.89).

### 3.4 The annotation of resistance genes differs depending on the contamination status of sequencing data

**Figure 4:**
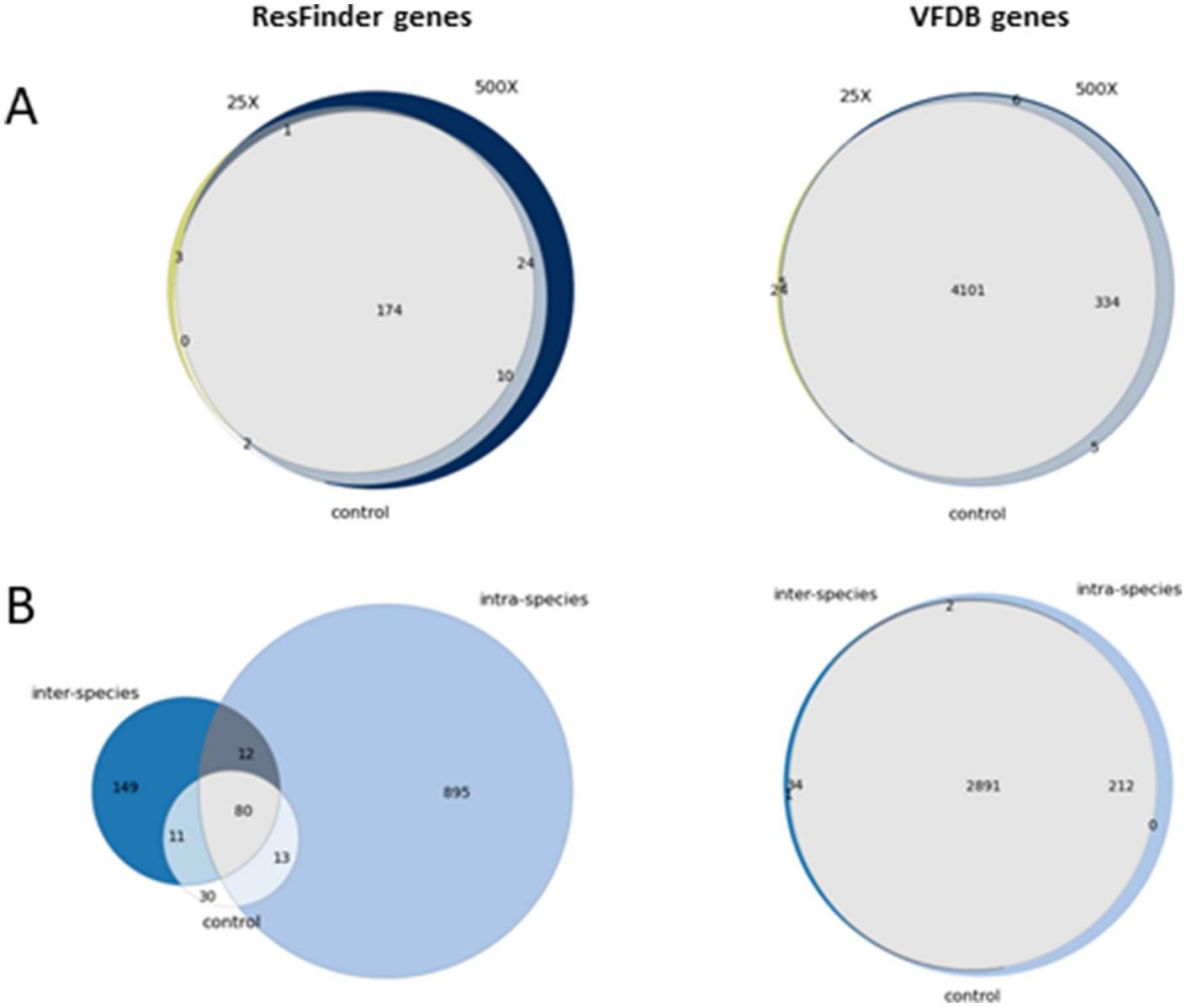
Impact of the coverage depth and contamination of raw reads on identification of virulence and resistance genes. Simulated reads with varying coverage depths and contamination types/levels were used to assess the detection of resistance genes (ResFinder) and virulence genes (VFDB). (A) To evaluate the effect of sequencing depth, gene identifications for each sample were individually compared with those of the data with a coverage depth of 25×, 100× (control), and 500×. (B) To assess the impact of contamination, gene identifications in intra-species, inter-species, and controls were compared to determine genes erroneously added or missed due to contamination for each sample.

As expected, fragmented and incomplete assemblies obtained at a low coverage depth (25×) show a significant decrease in the number of CDSs annotated by Bakta in 9/11 species, as well as a decrease in rRNA or tRNA genes in, respectively, 9/11 and 11/11 species (Fig. 4, available as supplementary data at *Bioinformatics Advances* online). Greater depths (250× and 500×) did not improve annotation compared with 100×. However, regarding the targeted annotation of virulence (i.e. ABRicate using the Virulence Factor Database, VFDB) and resistance (i.e. ResFinder) genes, a low depth of coverage did not affect detection, but a greater one increased the number of targeted annotated resistance genes (Fig. 4).

**Figure 5:**
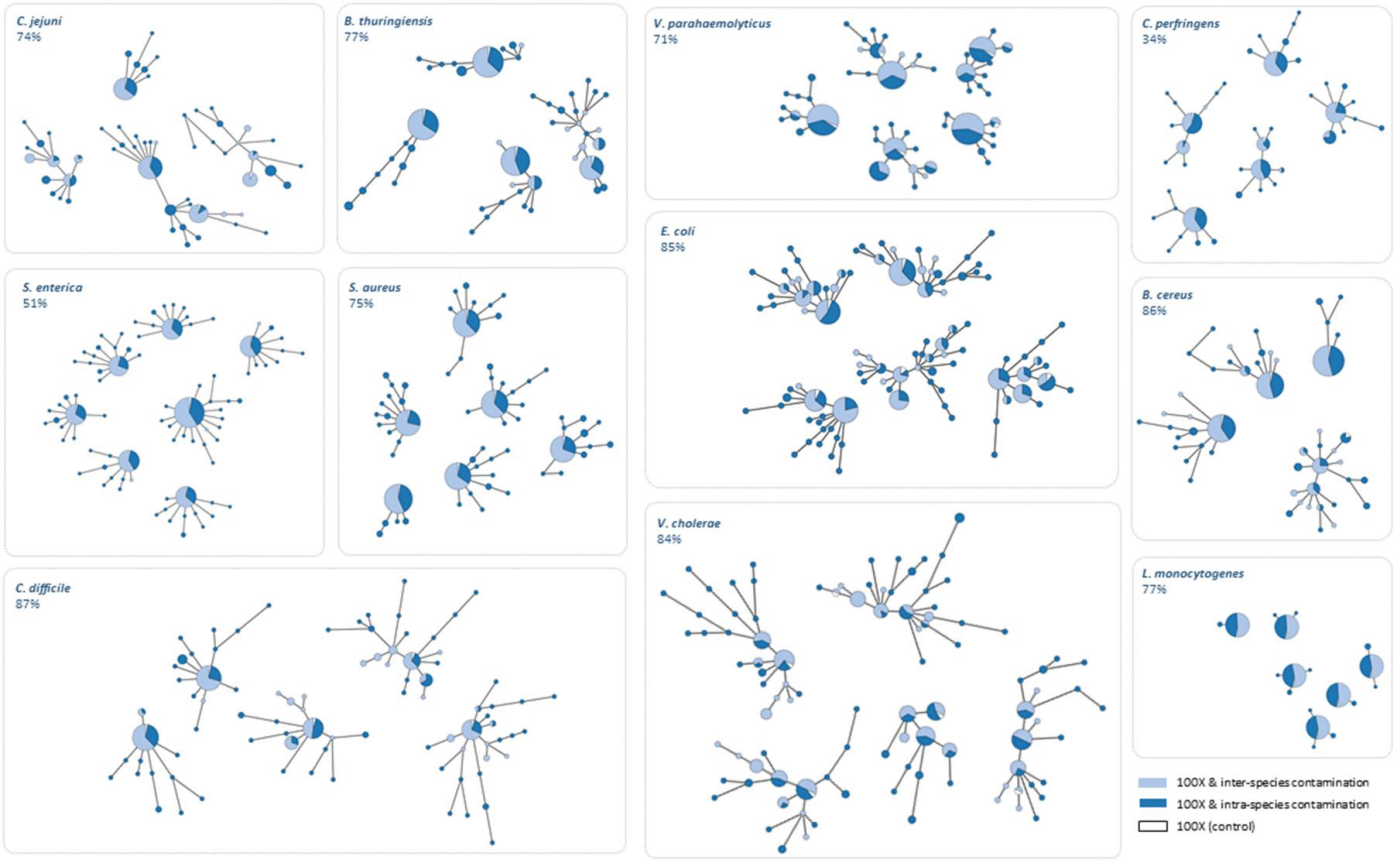
Impact of the contamination of raw reads on cgMLST clustering and distance between similar strains. All contaminated strains and 100× controls were compared by computing allelic distances on a restricted schema containing only genes found in all the samples. Completeness is provided for each analysis. Clusters shown in the figure correspond to the same initial reference genome: white boxes represent the control (simulation of reads at 100×), while dark and light blue pies represent, respectively, intra- and inter-species contaminated samples. Distances were evaluated in the complete schema by excluding genes not uniformly found in all the samples, and completeness of each analysis is displayed as a percentage for each species. Spanning trees were obtained using GrapeTree.

**Figure 6:**
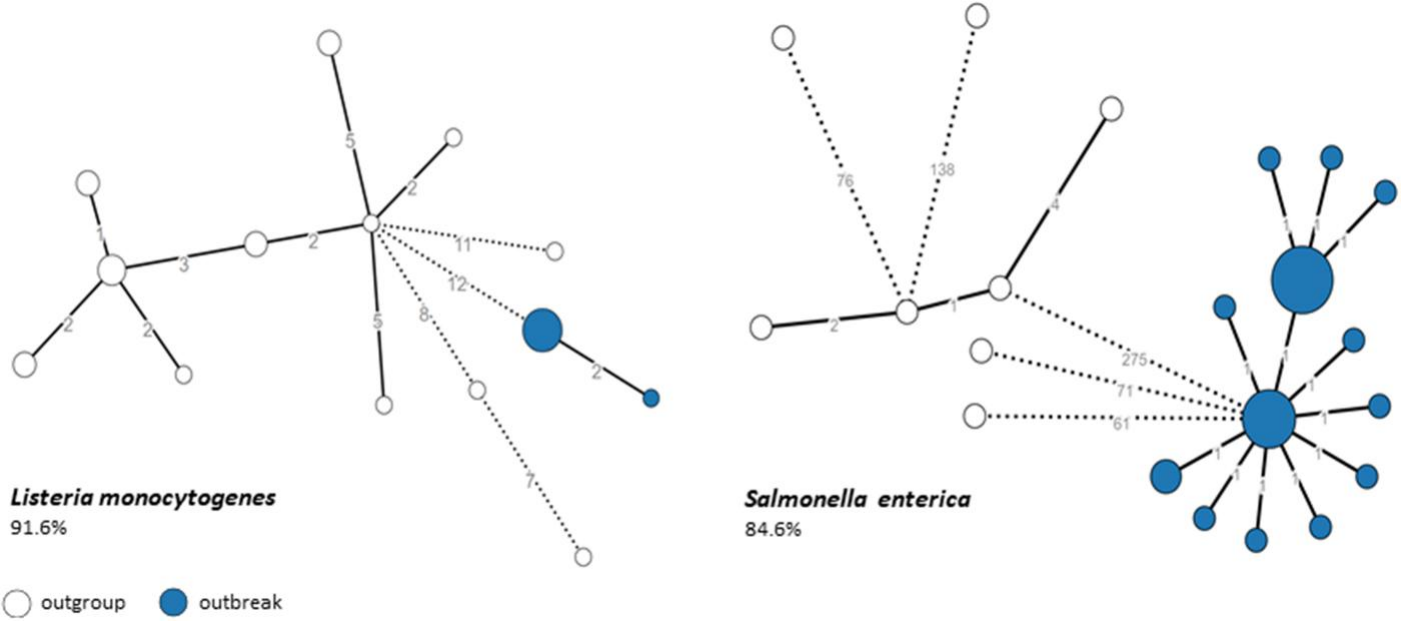
Application of BacWORK to the cgMLST clustering of two outbreaks. Two datasets from published outbreaks were downloaded, and the reads were analyzed using BacWORK. The cgMLST profiles obtained for each outbreak were compared against computed allelic distances between strains for genes found in all the samples. Spanning trees were obtained using GrapeTree. The resulting clustering is shown for the *Listeria monocytogenes* outbreak (23 samples) and the *Salmonella enterica* outbreak (34 samples). Blue pies correspond to strains involved in the outbreaks, while white pies correspond to outgroup strains. Schema completeness is displayed for both analyses.

Inter-species contamination did not impact genome annotation while intra-species contamination slightly impacted genome annotation, particularly the count of rRNAs in 10/11 species and tRNAs in 4/11 species (Fig. 4, available as supplementary data at *Bioinformatics Advances* online). Even for intra-species contamination, we did not find any link between the contamination ratio and genome annotation (Fig. 5, available as supplementary data at *Bioinformatics Advances* online). Targeted annotation of resistance genes is greatly affected, with gene annotation differing in contaminated samples from the controls. The impact of this is even greater for intra-species contamination, even at a low contamination ratio (Fig. 4 and Fig. 6, available as supplementary data at *Bioinformatics Advances* online). Indeed, we observed that a median of four more resistance genes was identified in contaminated samples. On the other hand, fewer virulence genes were found to differ in contaminated samples, with a median of one less gene detected (Fig. 4).

### 3.5 Contamination of sequencing data reduces accuracy of typing and serotyping

A low depth of coverage has a major impact on ST results. Indeed, at 25×, ST was missing for most samples (54/93) in all the species tested. At 50×, ST was still missing in a single sample. More importantly, ST was wrongly reported for two *V. parahaemolyticus* samples and one *C. jejuni* sample. At a greater coverage depth, ST was always reported with high accuracy. Some species-specific typing methods were affected by low-depth sequencing. We observed errors in serotyping of 4/11 *Salmonella enterica* samples. Spa type determination was wrongly reported in 2/9 *S. aureus* samples and missed in 1/9. At 50×, all typing results were similar to those of 100× simulated controls (Table 1).

The impact of inter-species contamination on ST results was minimal, with only two contaminated samples being returned as an undetermined ST. The impact of inter-species contamination on sequence type (ST) typing was also minor. However, unlike inter-species contamination, which occasionally returned undetermined STs, intra-species contamination led to errors in ST determination in three *C. jejuni* isolates (contamination ratio of 15%), four *C. difficile* isolates (contamination ratio of 13%), and one *S. aureus* isolate (contamination ratio of 14%) (Table 2). Interestingly, for both *C. jejuni* and *C. difficile*, the ST error involved the same cross-contaminated genomes. Species-specific typing methods remained largely unaffected by inter-species contamination, with just one inconsistency reported in SISTR serotyping for a contaminated *S. enterica* sample. The same results were observed for intra-species contamination, with more but still few mistakes being reported. For *E. coli*, three out of 200 samples tested were reported as STEC, whereas the expected result from an uncontaminated control was non-STEC. Contamination ratios were high (over 10%) in all the impacted samples and involved the same genome used for contamination. The spa type was wrongly reported for four *S. aureus* samples contaminated at 5% and 8%, involving two different genomes used for contamination purposes. Finally, the serotyping of *Salmonella enterica* was also impacted, five errors being reported for both SISTR and SeqSero2 tools, involving a total of six different samples. Errors reported by both tools were almost identical, and were the result of a high ratio of contamination (over 10%) by a genome of the same or a different species, depending on the case.

**Table 2:** Impact of contamination on typing approaches.^a^

Species	Typing method	Total samples	Errors in intra-sp.	Errors in inter-sp.
**All**	ST	2900	80	20
***Bacillus cereus***	Bt cluster	200	1 (1)	0
***Bacillus cereus***	Taxon	200	0	0
***Bacillus cereus***	Group	200	0	0
***Escherichia coli***	STEC	250	30	0
***Staphylococcus aureus***	Spa typing	300	4 (3)	0
***Salmonella enterica***	Seqsero2	400	50	0
***Salmonella enterica***	SISTR	400	50	10

a Simulated inter- and intra-species contaminated samples were typed and the results were compared against uncontaminated controls. Errors in sequencing typing results are displayed, with numbers in brackets corresponding to typing errors in samples not identified as contaminated by BacWORK. (intra-sp.: intra-species contamination; inter-sp.: inter-species contamination).

Reassuringly, ST typing errors can be easily avoided because contaminated samples were identified upstream by BacWORK (Table 2). *Salmonella enterica* serotyping errors were also all identified by the contamination filtering integrated in BacWORK, as were *E. coli* errors in STEC identification. For *S. aureus* and *B. cereus*, typing errors were not avoidable in 3/4 spa typing and 1/1 *B. thuringiensis* (Bt) cluster identification, as no in silico contamination of samples was detected by our tool.

Regarding core-genome typing, we observed that completeness increased with sequencing depth, rising from 30% at 25× to more than 90% at 50× and more (Fig. 7, available as supplementary data at *Bioinformatics Advances* online). We did not observe any differences in completeness with an increase in sequencing depth of between 50× and 500×. On the contrary, while contamination did not impact cgMLST completeness, it significantly changed allelic differences between strains (Fig. 5). The impact was slightly different regarding species with few allelic differences for *L. monocytogenes* and most *Bacillus* samples. *C. difficile*, *E. coli*, and *V. cholera* were the most affected by contamination, resulting in marked disparity in allelic differences between the control and contaminated strains (Fig. 8, available as supplementary data at *Bioinformatics Advances* online). Finally, we observed a strong correlation between the simulated contamination ratio and an increase in allelic differences between contaminated strains, whether intra-species or inter-species, compared with their controls (Fig. 9, available as supplementary data at *Bioinformatics Advances* online).

### 3.6 Runtime and storage consumption

To evaluate BacWORK’s performance and evaluate its scalability, the 75× datasets (five to eight samples per species, corresponding to a dataset ranging from 0.7 Go to 2.6 Go) were analyzed on a server with 192 threads (Intel Xeon Gold 6348H CPU @ 2.30 GHz) and 755 GB of RAM. The analyses were performed for each species and simulated depth independently, either using a SATA interface with remote NAS or internal NVMe drives on the server. Analyses on NVMe disks were 1.2 times faster than analyses run using SATA drives, as analyses on NVMe disks were completed in an average of 32 minutes, compared to 38 minutes on SATA drives. The most time-consuming steps were genome assembly and annotation, which substantially benefited from NVMe drives. The longest analysis steps were also the most storage-intensive, generating between 12 MB and 36 MB of data per sequenced strain, including the storage of trimmed and filtered reads but excluding raw data that are usually provided by sequencing platforms (Fig. 10, available as supplementary data at *Bioinformatics Advances* online). As for runtime, the required storage volume directly correlated with the length of the sequenced species’ genome.

### 3.7 Evaluation of reproducibility on real datasets

Genomic reproducibility is defined as the ability of a bioinformatics tool to maintain consistent results across technical replicates (Baykal *et al.* 2024). To assess the reproducibility of BacWORK, we evaluated our tool on public EFSA datasets for four pathogens: *C. jejuni*, *E. coli*, *L. monocytogenes*, and *S. enterica* (Fig. 11 and Table 2, available as supplementary data at *Bioinformatics Advances* online). The serotype and ST of *E. coli* strains were consistent in 10/10 and 9/10 strains, respectively. A single discrepancy was observed in one replicate of *E. coli* (i.e. serotype-:H11 instead of O26: H11), but no parameter could be identified to explain this error. Among sequencing replicates from *C. jejuni*, *L. monocytogenes*, and *S. enterica*, we observed that expected ST and serotype, if applicable, were conserved in all samples. As expected for in silico simulated samples, and as previously observed, replicates of *L. monocytogenes* are highly reproducible. Indeed, we did not observe any allelic differences between replicates. However, cgMLST profiles of technical sequencing replicates of *C. jejuni*, *L. monocytogenes*, and *S. enterica* were less reproducible. For *C. jejuni* and *S. enterica*, replicates varied between 0 and 8 allelic differences between strains, with a median of 1. *E. coli* strains were less reproducible, as observed on simulated data, with between 0 and 22 allelic differences observed between replicates (data not shown).

### 3.8 Application to outbreak investigations

To validate our approach, we applied BacWORK to data obtained during two separate foodborne outbreaks, one involving *L. monocytogenes* and the other, *S. enterica* (Cherchame *et al.* 2022, Halbedel *et al.* 2023). To avoid overestimation, allelic distance was estimated for both outbreaks on cgMLST genes found in all the samples. BacWORK results showed a cgMLST completeness of 91.6% for the 23 *L. monocytogenes* strains and 84.6% for the 34 *S. enterica* strains. Allelic distance clustered related strains into a single cluster for both outbreaks, significantly distant from outgroup strains used as controls (Fig. 6 and Table 3, available as supplementary data at *Bioinformatics Advances* online).

To confirm the importance of the original quality of the sequenced data prior to outbreak investigation, we performed a second analysis integrating samples of doubtful quality. We first contaminated four outbreak-related strains by mixing reads from another dataset at 5% and 10% (Fig. 12, available as supplementary data at *Bioinformatics Advances* online). For the *S. enterica* outbreak, one out of the four poor-quality strains appeared distant (five alleles) from the uncontaminated strains. Moreover, the addition of contamination into originally contamination-free strains decreased cgMLST completeness from 84.6% to 68%. For *L. monocytogenes*, as observed previously on simulated data, contamination did not have an impact on either allelic distance between strains or cgMLST completeness. In both outbreaks, BacWORK identified all the strains contaminated on purpose, which means that they should therefore be excluded from cgMLST analysis. This result confirmed the robustness of our approach in the case of outbreak investigations.

## 4 Discussion

The development of WGS-based surveillance platforms that centralize data in a unique storage system holds significant promise for global surveillance strategies. However, the success of such platforms relies upon the sharing of high-quality data and the establishment of robust quality thresholds. These are essential for ensuring reproducibility and repeatability of results across public health agencies. As we showed in this study, sharing genomes or cgMLST profiles derived from low-quality data can lead to sequence typing errors and missed opportunities for cluster detection, thereby compromising surveillance efforts. To detect these quality issues, we developed BacWORK, a robust and scalable workflow designed to analyze the sequencing of bacterial isolates. As presented in this study, BacWORK has been validated for 11 major pathogens involved in foodborne diseases. This bioanalyst-friendly workflow provides specific annotations for the most important pathogens in food outbreaks and integrates systematic screening for phages and plasmid signatures, enhancing genome annotations. BacWORK extends systematic screening for species-specific typing implemented in other workflows. It integrates modules for virulence gene detection with chromosomal or plasmid localization. BacWORK also incorporates spa typing for *S. aureus* (e.g. via Naura), more refined serotyping of *S. enterica* Typhimurium using an in-house *in silico* PCR approach, as well as the detection of *Campylobacter* host-specific markers (Thépault *et al.* 2017).

A recent study conducted by the BeONE consortium evaluated the concordance of WGS analyses across different WGS pipelines and showed relatively good congruence between tools (Mixão *et al.* 2025). In our study, we highlight important considerations for ensuring strict quality control and reproducibility of results by presenting an updated workflow developed according to best reproducibility practices. More importantly, we discuss potential reasons for discrepancies between pipelines by highlighting certain limitations in the detection of variations related to input data properties. To ensure accurate and relevant analyses, we implemented a detailed sample quality control module that flags low-quality inputs such as low depth of coverage or contaminated datasets. For instance, we observed that assemblies from low-depth datasets (e.g. 25×) were more fragmented, which in turn led to a higher rate of missed genes during de novo annotation or targeted gene searches (e.g. resistance or virulence genes). We also observed that highly fragmented assemblies tend to lead to more sequence typing errors. These findings underscore the importance of sufficient sequencing depth to ensure accurate and reliable genomic data for all pathogens and to discard insufficiently covered samples. Among the main workflows publicly available, few provide guidelines on minimum quality filtering requirements for WGS data. AQUAMIS, ProkEvo, TORMES, and ASA3P (Quijada *et al.* 2019, Schwengers *et al.* 2020, Deneke *et al.* 2021, Pavlovikj *et al.* 2021) apply a filter based on the number of contigs (i.e. 100 to 300 contigs depending on the pathogen), but they do not discuss minimum sequencing depth. Bactopia and CABGen (Duré *et al.* 2022) do not propose any quality thresholds, while rMAP (Sserwadda and Mboowa 2021) has been deliberately designed not to include filtering steps that could result in the loss of essential genes. As data filtering mechanically reduces sequencing depth and is associated with gene loss, we tend to agree with this approach. However, a previous study showed that including low-quality reads can be counterproductive, resulting in poorer gene annotation (Merda *et al.* 2024). In line with EFSA and FDA recommendations (Timme *et al.* 2020, EFSA Report 2021), AQUAMIS (Deneke *et al.* 2021) and Nullarbor (Seemann 2015) propose a variable minimum sequencing depth of between 20× and 40× depending on the pathogen. However, our results, which are consistent with our previous study, indicate that cgMLST completeness remains poor at sequencing depths between 25× and 50×. Using such incomplete data may compromise the accuracy of clustering analyses, particularly in the context of outbreak investigations. Furthermore, at these depths, missing genes were also observed in both targeted virulence gene annotation and global CDS annotation. We furthermore observed that annotating resistance genes directly on the reads rather than on the assembly overcomes this limitation.

In addition to sequencing depth, we also investigated the impact of low-level contamination on sequencing results. While several studies have already assessed the performance of publicly available tools for detecting high levels of contamination, little is known about their sensitivity at lower contamination levels. For example, Pightling *et al.* (2019), reported that intra-species contamination causes errors in strain clustering, while inter-species contamination had less impact on clustering. In AQUAMIS, authors also studied the impact on results of contamination during sequencing. They observed that at a high level of contamination (above 10%) assemblies are mostly affected by higher fragmentation and duplication (Deneke *et al.* 2021). Within the BacWORK validation module, we focused on the impact of low-level contamination, as it is harder to detect but equally important. We previously observed that even state-of-the-art detection tools may fail to detect WGS contamination, especially when the contaminant is present at a low ratio (<2%). Indeed, even if the precision of these tools is satisfying even at low ratios, recall too can be low. This is particularly true for some foodborne pathogen species, thus leading to missed contaminated samples and potentially erroneous results. Contamination detection can be challenging (Cornet and Baurain 2022), and the development of new tools based on various detection strategies could help to reduce the number of undetected contaminated samples. Indeed, we found that undetected contaminated samples could pose significant challenges to genomic surveillance, as even low-level contamination can lead to fragmented assemblies and typing errors. Therefore, users should be caution when interpreting assembly quality metrics, since even slight deviations from expected values may indicate a potential contamination event. Contamination does not appear to have an impact on CDS identification, but further investigations are required to ensure that genes identified by tools like Bakta are consistent across control and contaminated samples. Apart from the AQUAMIS (Deneke *et al.* 2021) workflow, there are few recommendations on contamination detection in WGS data in either the EFSA or FDA guidelines (Timme *et al.* 2020, EFSA report 2021). However, systematically integrating such quality control steps could be crucial.

More than 5% of public fastq files of sequenced and deposited foodborne outbreak pathogens appear to show signs of contamination (Pightling *et al.* 2019). Given the varying sensitivity of available tools, this can pose challenges when using public datasets for research or surveillance on bacterial foodborne diseases, particularly regarding the sensitive questions of resistome and virulome characterization (Cobo-Díaz *et al.* 2021). We demonstrate that even low levels of contamination can fragment assemblies and significantly affect resistome characterization, posing challenges for genomic surveillance. Thus, high-quality data are shown to be essential for accurate annotation and antimicrobial resistance (AMR) gene detection to avoid an over- or underestimation of multi-resistance loci in screened pathogen populations (Cobo-Díaz *et al.* 2021).

Finally, with the rapid progress in sequencing technologies and the exponential growth of genomic databases, it is becoming increasingly important to consider not only the quality and interoperability of the data, but also their environmental impact. The carbon footprint from storing WGS data is of particular concern. Estimates suggest that sequencing 1000 strains at a minimal required depth (75×) on a cloud-equivalent system can produce 3–8 kg of CO_2_. By 2030, assuming linear growth, the carbon footprint could reach 21–56 kg of CO_2_. Under an exponential growth scenario, and as WGS becomes more widespread, the carbon impact could escalate to 0.3–1 ton of CO_2_. This highlights the need for strategic data management, focusing on which data should be retained mid- and long-term, considering the rapid evolution of sequencing technologies and analysis algorithms. Indeed, estimations suggest that genomic data will reach one trillion gigabytes by 2025 (GenBank and WGS Statistics), emphasizing the exponential growth of data in repositories like GenBank. The use of standardized workflows like BacWORK, developed in accordance with FAIR principles, ensure the reliability and usability of these data and should further facilitate optimal data storage and minimal environmental impact while maximizing the utility of genomic surveillance data. BacWORK provides easily accessible outputs by summarizing sequencing results into gene profiles and cgMLST data, allowing for rapid comparison between pathogens. It provides standardized results over time and avoids redundant analysis by using Mamba and Snakemake to ensure accessibility and reproducibility of results. Finally, the interoperability of outputs is enhanced through the use of state-of-the-art tools designed for major pathogens, ensuring compatibility with common versions and databases.

In conclusion, this work has highlighted the critical importance of quality control for WGS data analysis of pathogenic foodborne bacterial strains. We report that even low levels of contamination or insufficient sequencing depth can significantly impact key results, such as bacterial typing and annotation, and we have addressed these challenges by developing BacWORK (https://github.com/FBi-ANSES/BacWORK), a workflow that integrates multiple steps for the analysis and interpretation of WGS results from bacterial isolates.

## Supplementary Material

vbag130_Supplementary_Data

## Data Availability

The source code for BacWORK is available at GitHub (https://github.com/FBi-ANSES/BacWORK) and all simulated datasets are available on demand (>1.5 TB).
