# Supplementary material for: Impact of sequencing read quality on whole-genome sequencing outcomes for foodborne pathogens using a BacWORK standardized analysis workflow
Source: Bioinform Adv. 2026 May 7;6(1):vbag130. doi: 10.1093/bioadv/vbag130 (PMC13265382; doi:10.1093/bioadv/vbag130)
Supplement: vbag130_Supplementary_Data [file vbag130_supplementary_data.zip › Additional_file.pdf]

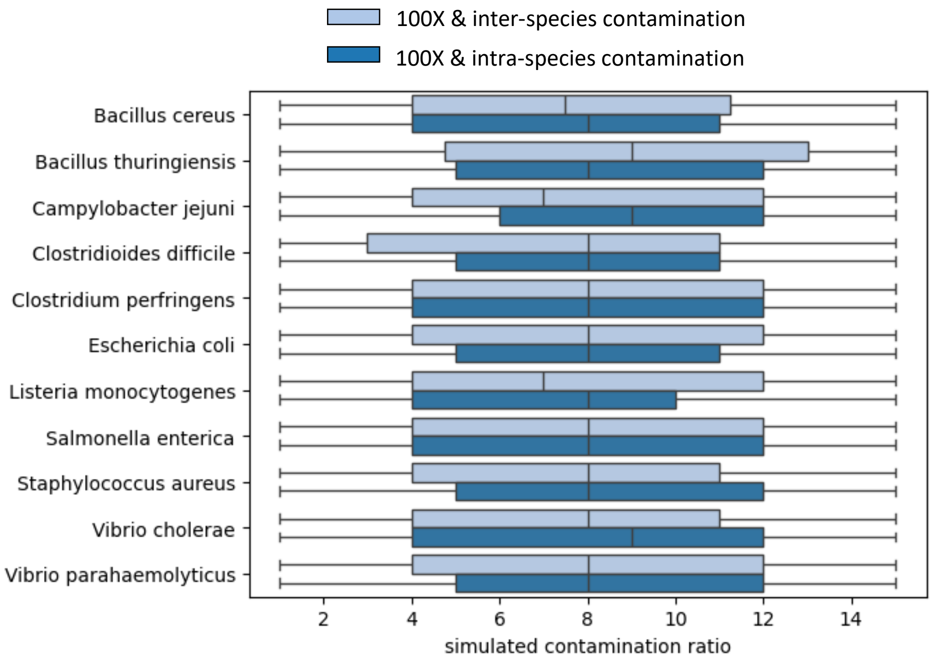

Supplementary figure 1: Distribution of simulated contamination ratio. For each 100X simulated dataset (i.e., pairs of short reads), additional reads from either another species (inter-species contamination) or the same species (intra-species contamination) were randomly chosen as contaminants. The contamination ratio was also randomly selected, ranging from 1% to 15%, to simulate randomly contaminated samples for each species and type of contamination.

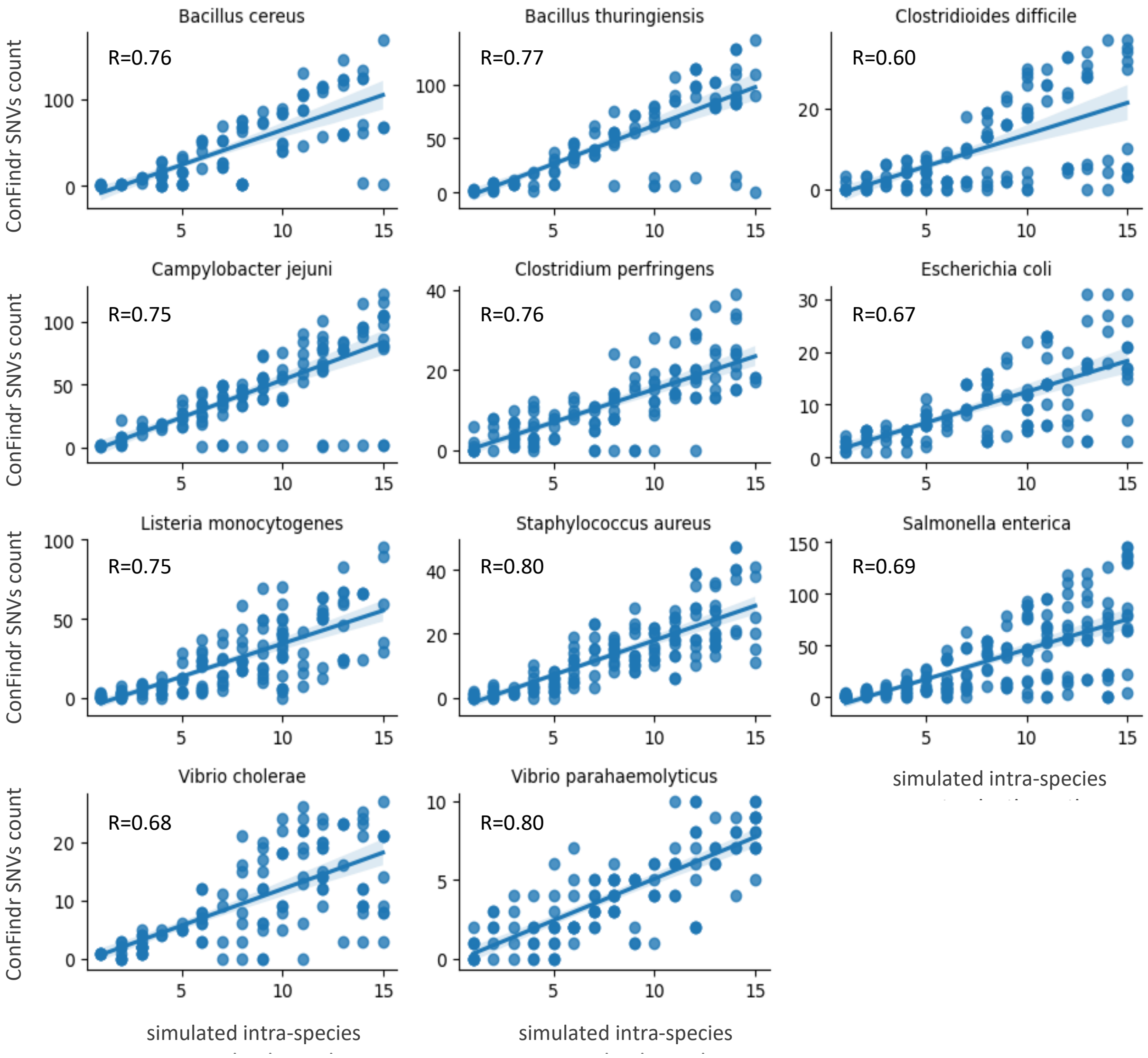

Supplementary figure 2: Correlation between simulated contamination ratio and SNVs detected by ConFindr. For each intra-species contaminated samples (i.e., pairs of short reads artificially contaminated within species), ConFindr was used to output SNVs that point for contamination. Correlation (R) between detected SNVs using rMLST database and expected contaminated ratio was computed for each species.

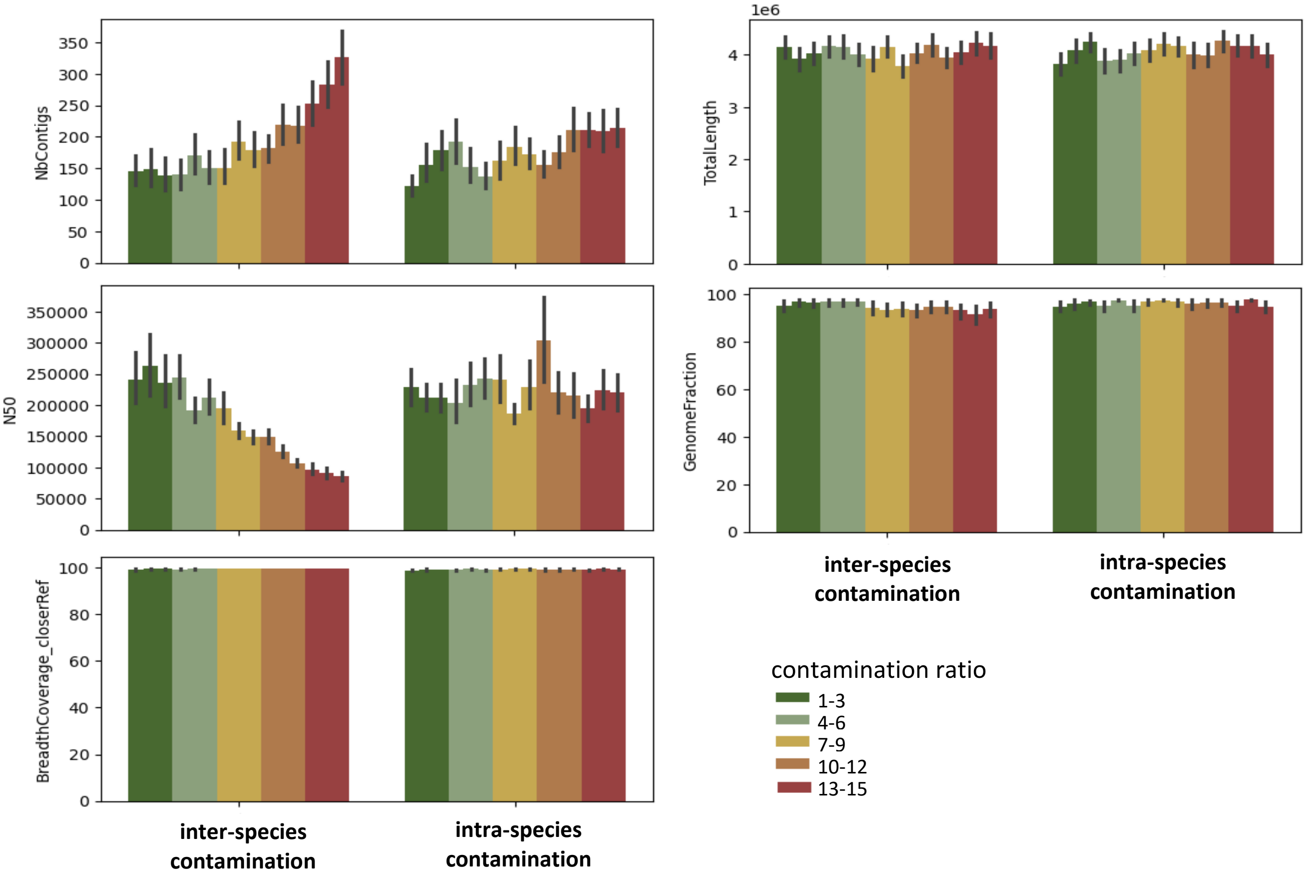

Supplementary figure 3: Impact of the contamination ratio of raw reads on assembly. Simulated reads with various depths and contamination types and levels were assembled with BacWORK. The impact of reads quality on assembly was evaluated using Breadth Coverage, Genome Fraction, N50, Number of Contigs, and Total Length of Assembly metrics for each simulated contamination range.

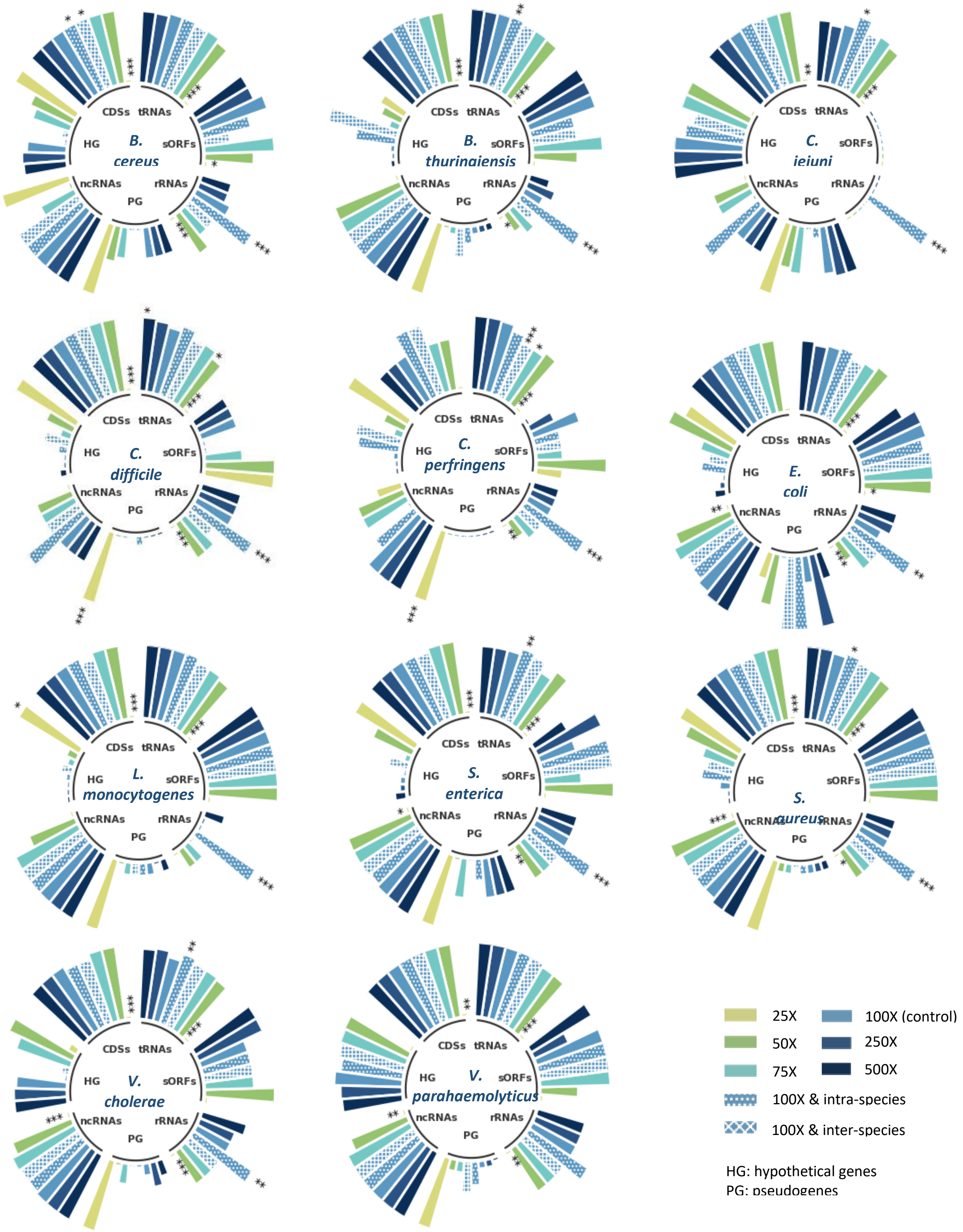

Supplementary figure 4: Impact of the coverage depth and contamination of raw reads on annotation of assembly. Assemblies obtained from simulated reads with various depth and contamination types and levels were annotated with BacWORK. The impact of reads quality on assembly annotation was evaluated using ncRNAs, rRNAs, CDSs, sORFs, hypotheticals'genes (HG) and pseudogenes (PG) counts. For the plots, median for each species were scaled using the min-max scaler

method. All conditions were compared using Student’s t-test with non-contaminated 100X used as control.

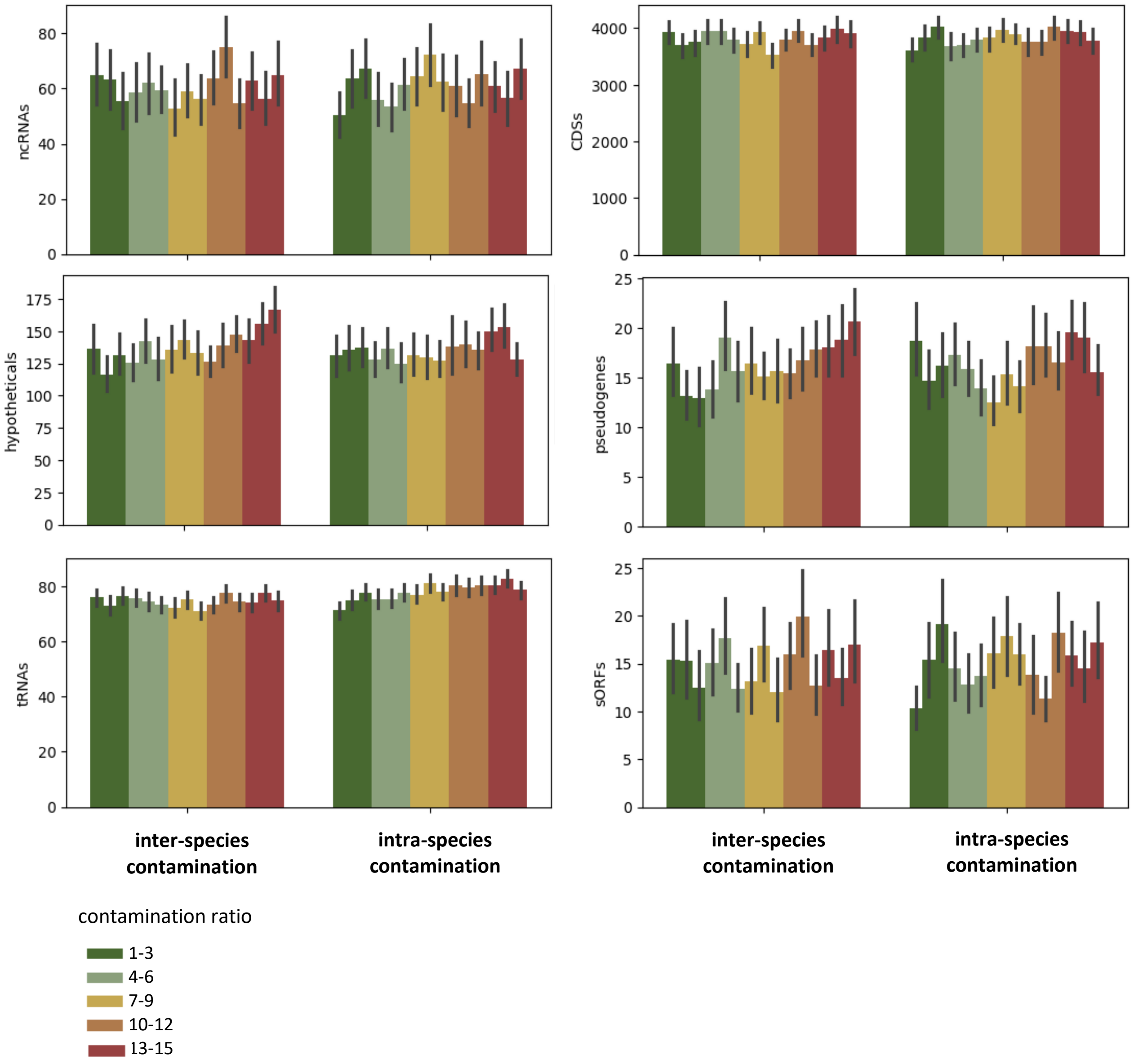

Supplementary figure 5: Impact of raw reads contamination ratio on annotation of assembly. Simulated reads with various coverage depths and contamination types and levels were assembled with BacWORK. The impact of reads quality on assembly annotation was evaluated using ncRNAs, rRNAs, CDSs, sORFs, hypotheticals'genes and pseudogenes counts for each simulated contamination range.

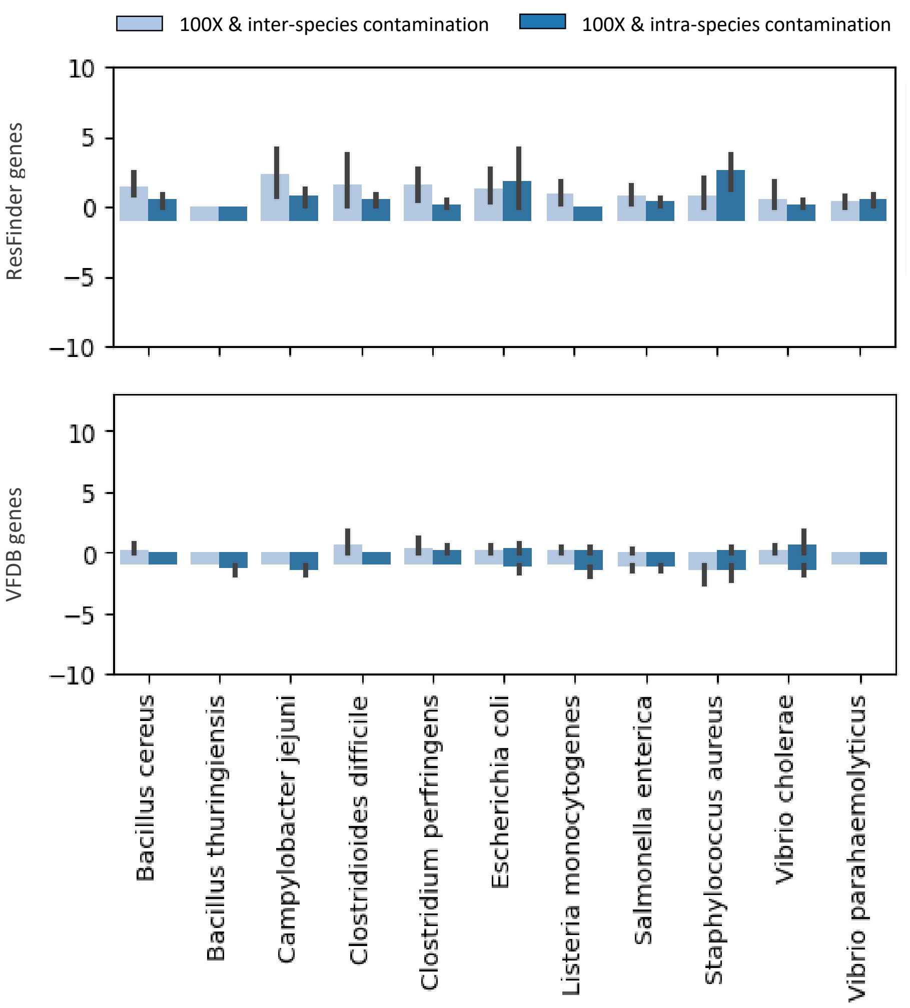

Supplementary figure 6: Impact of raw reads contamination on identification of virulence and resistance genes. Simulated reads with varying coverage depths and contamination types/levels were used to assess the detection of resistance genes (ResFinder) and virulence genes (VFDB). For each sample, the numbers of genes added or missed compared to uncontaminated samples were computed for both inter- and intra-species contaminations.

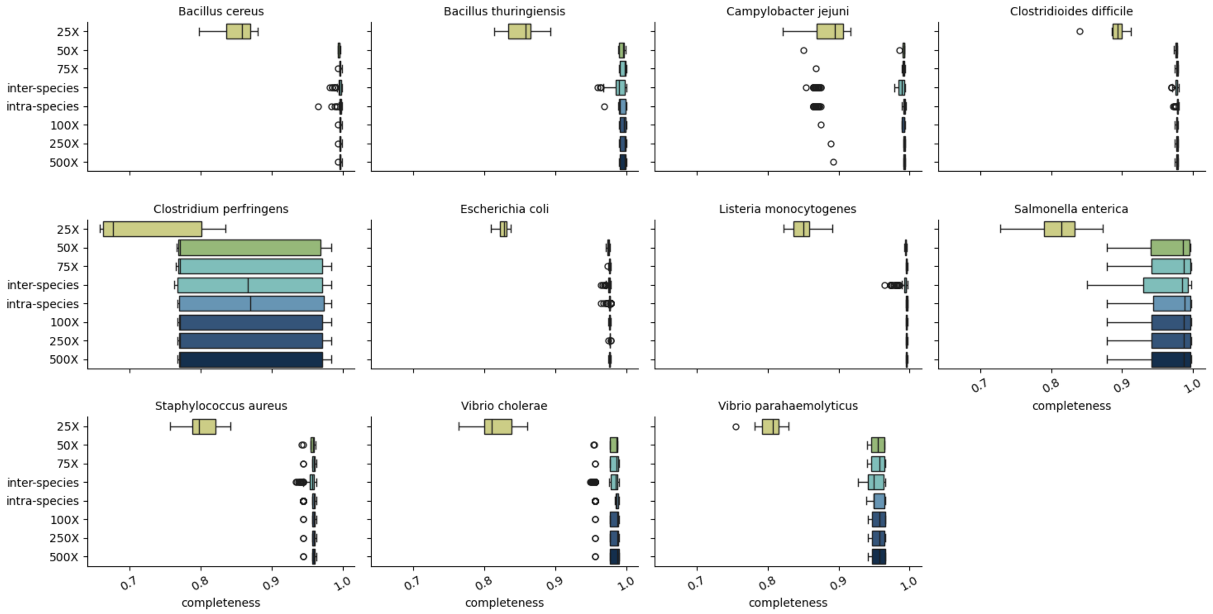

Supplementary figure 7: Impact of the coverage depth and contamination of raw reads on cgMLST completeness. Assemblies were typed using cgMLST approaches with chewBBACA integrated into the BacWORK workflow. The quality of cgMLST profiles was assessed by evaluating schema completeness (i.e., the fraction of core genes found in each sample).

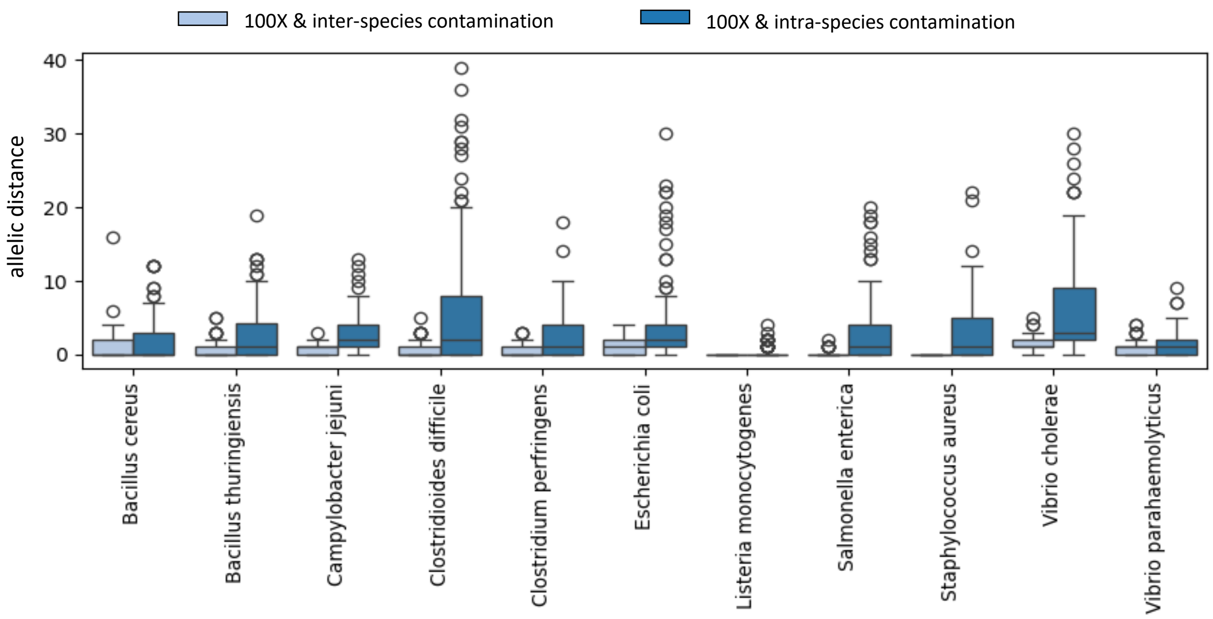

Supplementary figure 8 Impact of the contamination of raw reads on cgMLST estimated allelic distances. For each species, all inter- and intra-species contaminated strains were compared to 100X controls to evaluate allelic distances between samples. Distances were evaluated in the complete schema by excluding genes differently found in samples.

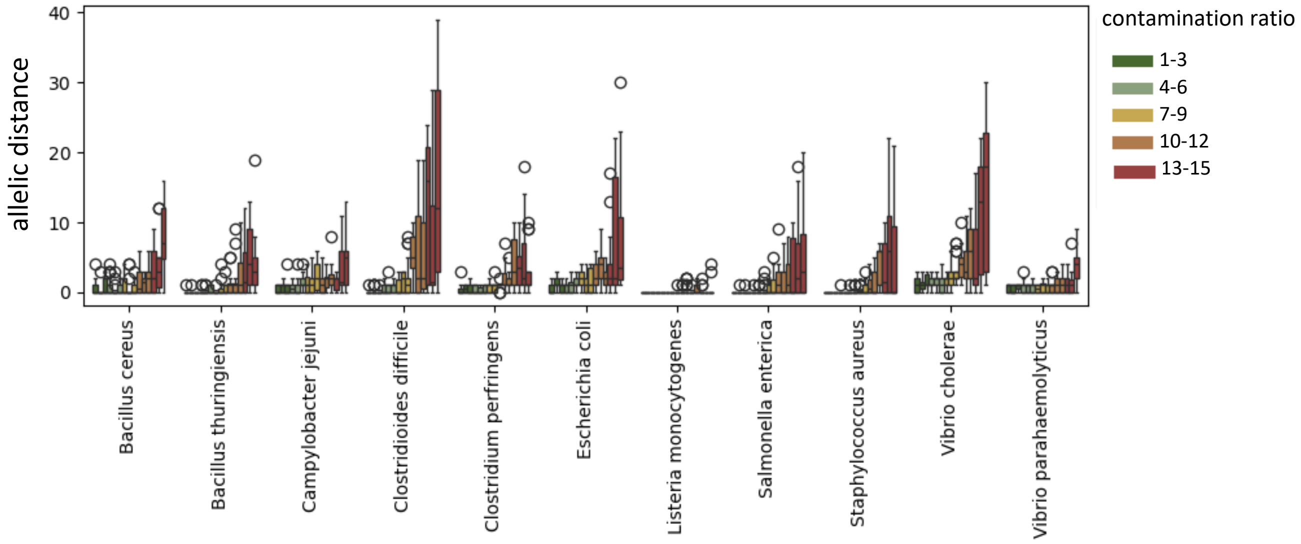

Supplementary figure 9: Impact of the contamination ratio of raw reads on cgMLST estimated allelic distances. For each species all simulated contaminated ratio were compared to 100X controls to compute allelic distances between samples. Distances were evaluated in the complete schema by excluding genes differently found in samples.

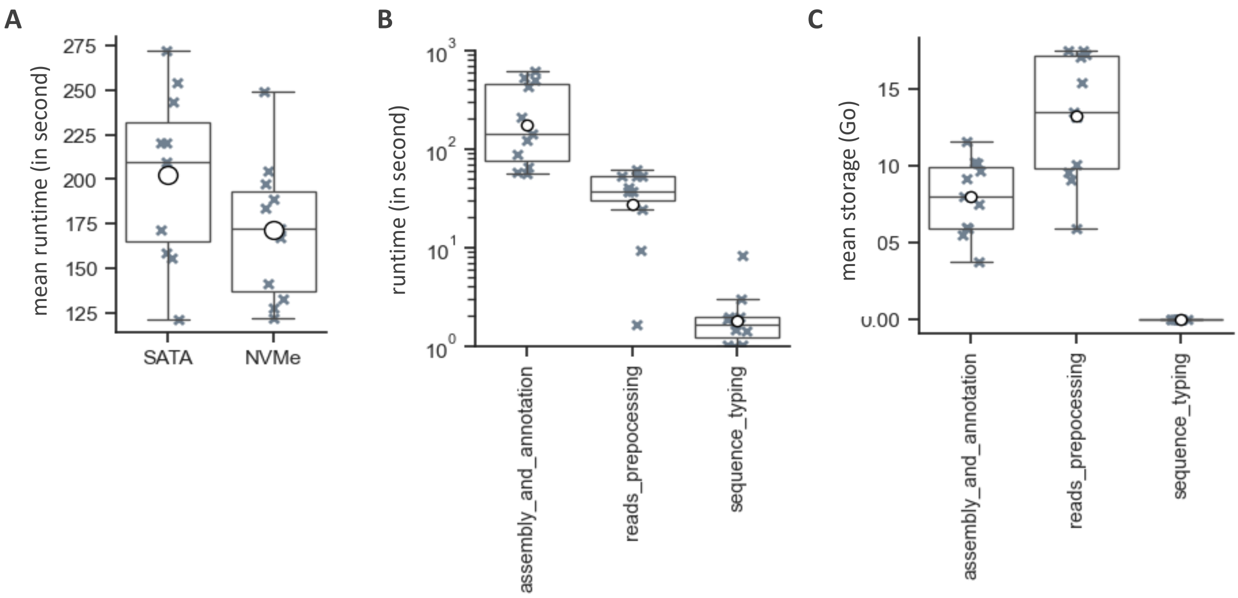

Supplementary figure 10: Overall performance of BacWORK. Runtime and amount of data produced by BacWORK were evaluated on 75X simulated samples across our selection of 11 species. Mean runtime per species was assessed using either NVMe scratch storage on the calculation server or an external SATA storage bay (A). Mean runtime per step (B) and required storage (C) per strain was also estimated to compare the main steps of the workflow. Species-specific typing runtime and outputs were not evaluated, as tools and number of methods vary for each of the 11 species.

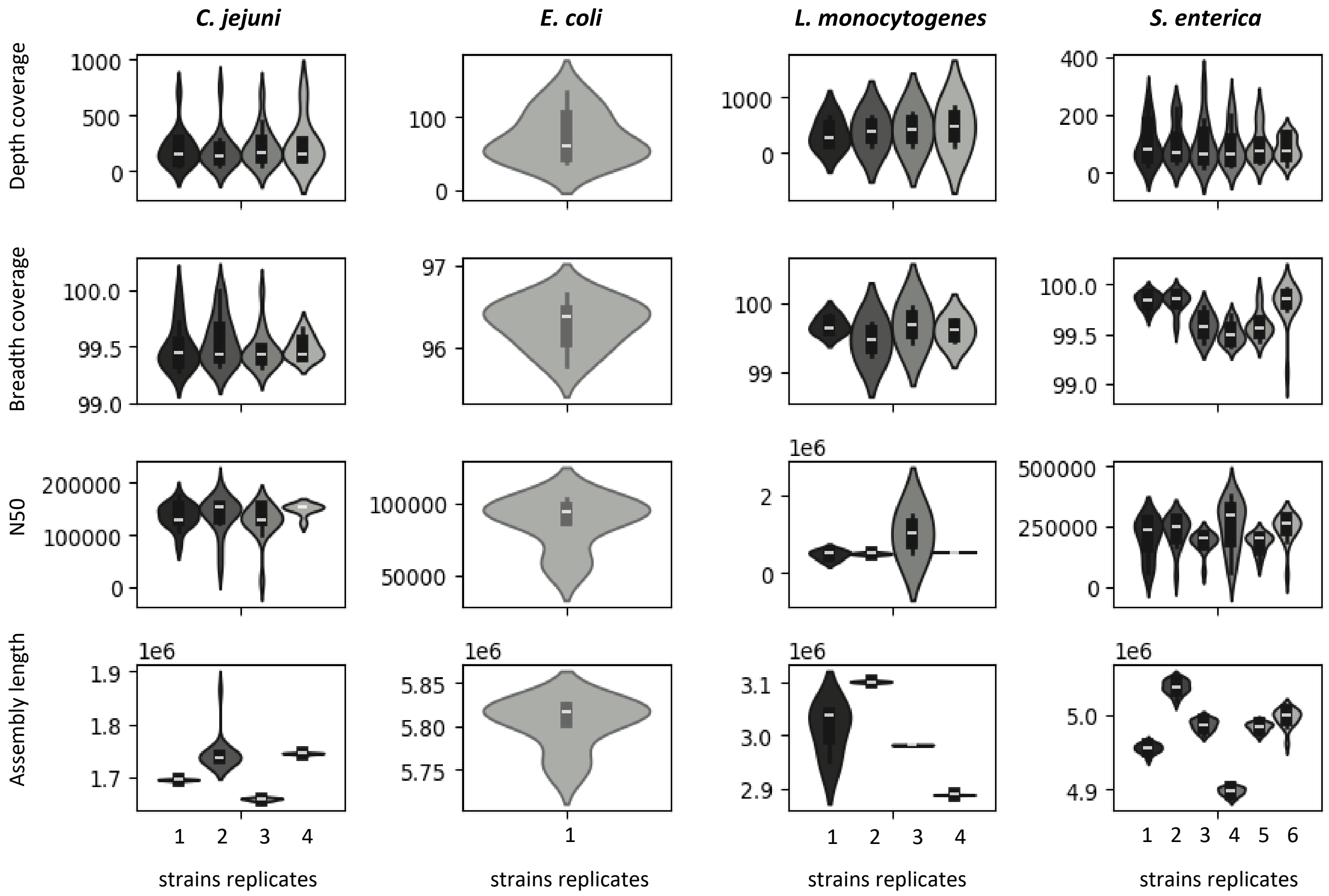

Supplementary figure 11: Description of EURLs datasets provided for *Campylobacter jejuni*, *Escherichia coli*, *Listeria monocytogenes* and *Salmonella enterica*. EURLs dataset were analyzed with BacWORK (9 *Escherichia coli* (1 strain), 58 *Campylobacter jejuni* (4 strains), 9 *Listeria monocytogenes* (4 strains) and 83 *Salmonella enterica* (6 strains)). Assembly resume per strains is displayed: for each species violin plots represent different replicates of a strain.

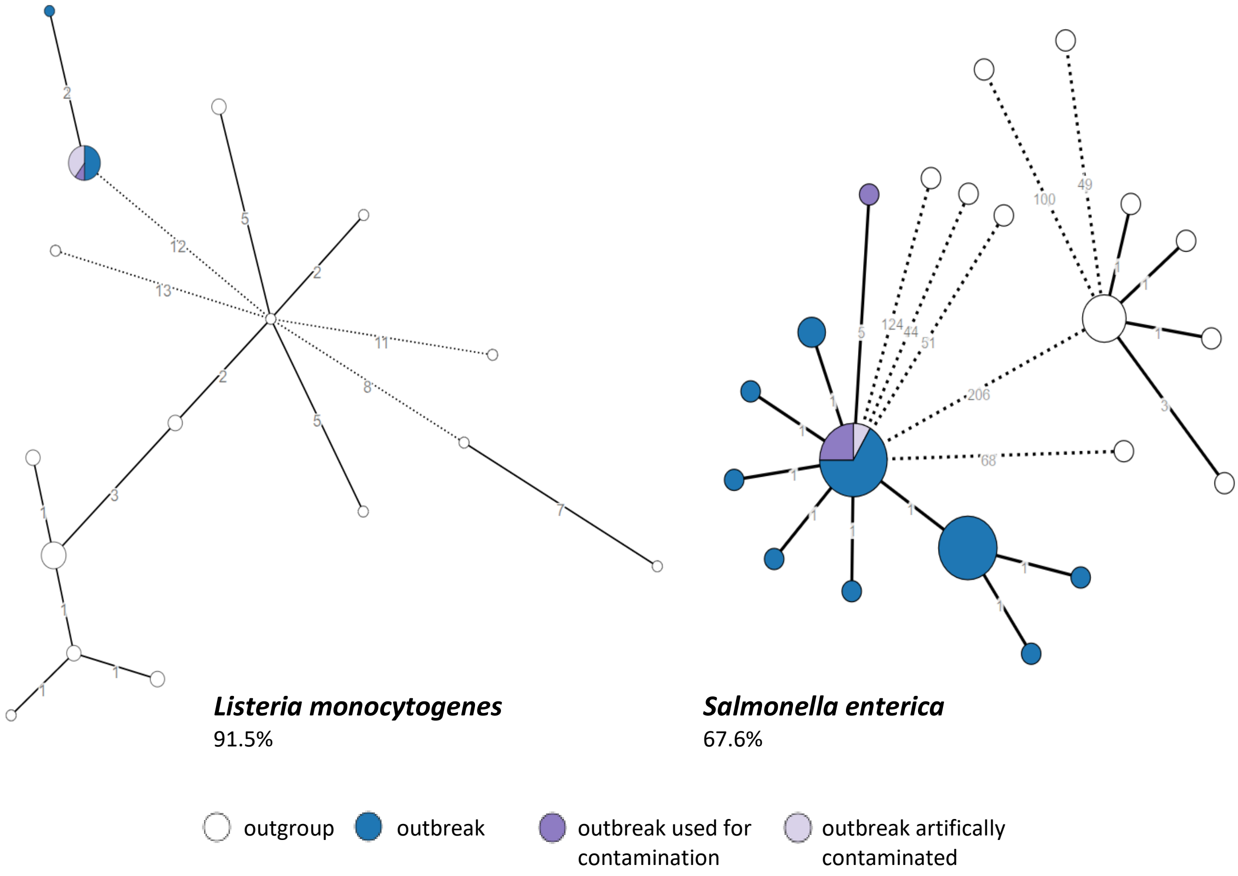

Supplementary figure 12: Application of BacWORK to the cgMLST clustering of two outbreaks artificially contaminated. Two datasets from published outbreaks were downloaded, and the reads were analyzed using BacWORK. For each outbreak a sample were artificially contaminated: 2 intra-species contamination à 5% and 10% and 2 inter-species-contamination at 5% and 10%. The obtained cgMLST profiles for each outbreak were compared to compute allelic distances between strains on genes found in all samples. Spanning tree were obtained using GrapeTree. The resulting clustering is shown for *Listeria monocytogenes* outbreak (23 samples) [57] and the *Salmonella enterica* outbreak (34 samples) [58]. Colored pies correspond to strains involved in the outbreaks, while white pies correspond to outgroup strains. Dark purple pie corresponds to outbreak sample used for contamination (raw sample), light purple pies to artificially contaminated outbreak samples and blues pies to other outbreak samples. Schema completeness is displayed for both analyses.

Supplementary tables legends

Supplementary Table 1: Comparison of BacWORK and existing workflows for bacterial isolates WGS. Tools available in the literature (AQUAMIS, Bactopia, Nullarbor, TORMES, ASA3P, rMAP, CABGen and ProkEvo) were compared to BacWORK.

Supplementary Table 2: BacWORK results summary for EURLs dataset. BacWORK summary output for all samples obtained from EURLs: 9 *Escherichia coli* (1 strain), 58 *Campylobacter jejuni* (4 strains), 9 *Listeria monocytogenes* (4 strains) and 83 *Salmonella enterica* (6 strains).

Supplementary Table 3: BacWORK results summary for outbreak dataset. BacWORK summary output for all samples obtained from 2 published outbreak dataset: 23 *Listeria monocytogenes* [59] and 34 *Salmonella enterica* [60].

Supplementary Table 4: List of tools, versions and parameters used in BacWORK validation.

Supplementary Table 5: cgMLST schema used in BacWORK.

Supplementary Table 6: Simulated dataset at different coverage depths. List of all simulated samples at 6 different coverage depths (25X, 50X, 75X, 100X, 250X and 500X) with accession numbers of original genomes.

Supplementary Table 7: Simulated contaminated dataset. List of all simulated samples with intra- and inter-species contaminations with accession numbers of original genomes and accession numbers of genomes used for contamination, as well as contamination ratio.
